# Developmental emergence of first- and higher-order thalamic neuron molecular identities

**DOI:** 10.1242/dev.202764

**Published:** 2024-09-30

**Authors:** Quentin Lo Giudice, Robin J. Wagener, Philipp Abe, Laura Frangeul, Denis Jabaudon

**Affiliations:** ^1^ Department of Basic Neurosciences, University of Geneva, 1202 Geneva, Switzerland; ^2^ NeuroNA Human Cellular Neuroscience Platform (HCNP), Fondation Campus Biotech Geneva, 1202 Geneva, Switzerland; ^3^ Clinic of Neurology, Geneva University Hospital, 1211 Geneva, Switzerland; ^4^ Université Paris Cité, Imagine Institute, 75015 Paris, France

**Keywords:** Brain development, Neuronal identity, Thalamus, First-order nuclei, Higher-order nuclei

## Abstract

The thalamus is organized into nuclei that have distinct input and output connectivities with the cortex. Whereas first-order (FO) nuclei – also called core nuclei – relay input from sensory organs on the body surface and project to primary cortical sensory areas, higher-order (HO) nuclei – matrix nuclei – instead receive their driver input from the cortex and project to secondary and associative areas within cortico-thalamo-cortical loops. Input-dependent processes have been shown to play a crucial role in the emergence of FO thalamic neuron identity from a ground-state HO neuron identity, yet how this identity emerges during development remains unknown. Here, using single-cell RNA sequencing of the developing mouse embryonic thalamus, we show that, although they are born together, HO neurons start differentiating earlier than FO neurons. Within the FO visual thalamus, postnatal peripheral input is crucial for the maturation of excitatory, but not inhibitory, neurons. Our findings reveal different differentiation tempos and input sensitivities of HO and FO neurons, and highlight neuron type-specific molecular differentiation programs in the developing thalamus.

## Teaser

**Summary:** Single-cell RNA sequencing reveals that first- and higher-order thalamic neurons have distinct differentiation dynamics and developmental sensitivities to peripheral input, highlighting neuron type-specific molecular differentiation programs in the developing thalamus.

## INTRODUCTION

The thalamus is most often referred to in relation to its function as a relay from sensory organs in the periphery *–* the retina, cochlea and skin mechanoreceptors *–* to their corresponding target area in the neocortex *–* the primary visual, auditory and somatosensory cortices (Clascá et al., 2012; López-Bendito and Molnár, 2003). Such relay function is mediated by so-called first-order (FO) thalamic nuclei, i.e. the dorsolateral geniculate nucleus (LG) for vision, the ventrobasal nucleus (VB) for somatosensation and the ventral medial geniculate nucleus (vMG) for audition.

**Fig. 1.**
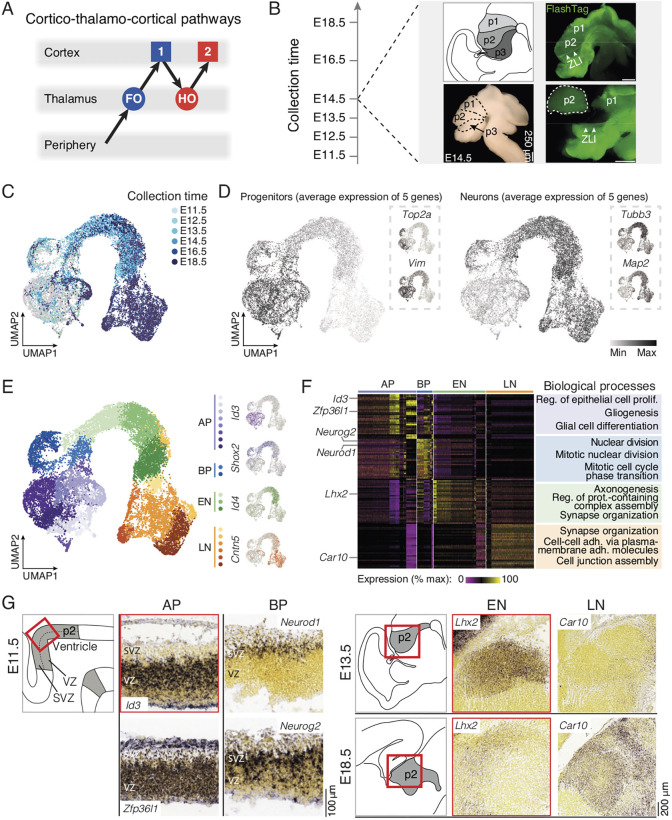
**Thalamic stage-dependent transcriptional diversity.** (A) Schematic representation of cortico-thalamo-cortical pathways. (B) Schematic illustration of the experimental timeline and the procedure for microdissection. (C) UMAP representation of the single-cell RNA sequencing dataset color coded by age of collection. (D) Progenitors and neurons can be distinguished by their combinatorial expression of key marker genes (*n*=5 transcripts with *Btg2*, *Pax6*, *Vim*, *Hes1* and *Nes* for progenitors; *Map2*, *Tubb3*, *Eno2*, *Rbfox3* and *Nefl* for neurons). (E) Cluster analysis reveals transcriptionally distinct and temporally dynamic cellular clusters (left): apical progenitor (AP) cells in purple gradient, basal progenitor (BP) cells in blue gradient, early neuron (EN) cells in green gradient and late neuron (LN) cells in orange gradient. Individual cell type representative genes are shown in their respective color (right). (F) Expression of the top 30 most expressed genes by cellular cluster highlight cellular diversity (left). Example of biological processes of gene ontologies associated with each cellular cluster. (G) Schematic representation of the p2 domain at E11.5, E13.5 and E18.5, and *in situ* hybridization sections of selected differentially expressed genes showing distinctive expression in AP, BP, EN and LN cells. Image credit: Allen Institute for Brain Science (https://developingmouse.brain-map.org/). FO, first order; HO, higher order; SVZ, subventricular zone; VZ, ventricular zone; ZLI, zona limitans intrathalamica. Scale bars: 250 μm in B; 100 μm in G (left); 200 μm in G (right).

Most of the thalamus, however, does not serve such functions, but instead acts within cortico-thalamo-cortical loops. In these loops, input from layer 5b neurons in primary sensory areas is forwarded to other cortical areas (secondary sensory areas and associative areas), allowing integration of sensory information and sensorimotor modulation (Antón-Bolaños et al., 2018; Clascá et al., 2012; Jabaudon, 2017; Theyel et al., 2009). As examples, the higher-order (HO) nucleus for vision is the latero-posterior nucleus (LP), while the posteromedial nucleus (Po) is the HO nucleus for somatosensation. Hence, for each sensory modality, a cortico-thalamo-cortical loop exists in which primary sensory cortex receives input from exteroceptive FO nucleus, and projects back to the corresponding HO thalamic nucleus, which then targets a secondary sensory cortex (Fig. 1A).

Thalamic neuron wiring is progressively established during embryonic and postnatal development. HO and FO neurons are born together around E11, because thalamic neurogenic gradients are organized in an outside-in pattern rather than along HO-FO divisions (Altman and Bayer, 1979 and https://neurobirth.org/). Axons from the hindbrain carrying sensory information from the periphery reach putative FO nuclei at E16, whereas the axons of the latter reach the cortex at E18.5, where they initially form transient synaptic connections with the subplate, before invading the cortex during the first few postnatal days (Erzurumlu and Gaspar, 2012; Guido, 2018; Huerga-Gómez et al., 2023; Martini et al., 2021; Pouchelon et al., 2012). Uncorrelated spontaneous activity is detectable within the thalamus as early as E12, whereas waves of spontaneous synchronic activity are present from E14 onwards (Moreno-Juan et al., 2017). Interestingly, spontaneous activity initially spreads across FO and HO nuclei (Moreno-Juan et al., 2017), suggesting that these thalamic regions have a common ontogeny.

Recent findings have identified the molecular identities of developing thalamic neurons and shed light on their clonal relationships, yet how this relates to the distinction between FO and HO neuron identity remains unclear (Gezelius et al., 2017; Govek et al., 2022; Moreno-Juan et al., 2017; Shi et al., 2017; Yuge et al., 2011). Input-dependent processes appear to play a role in this process as input ablation (e.g. vibrissectomy or enucleation) performed at critical periods of early postnatal development dramatically affects the development of FO thalamic nuclei and their corresponding primary cortical target (Frangeul et al., 2016; Giasafaki et al., 2022; Grant et al., 2016; Van der Loos and Woolsey, 1973). This is visible in terms of morphological organization, whereby infraorbital nerve section disrupts the somatotopic map of the whiskers in the VB (Frangeul et al., 2014, 2016; Jensen and Killackey, 1987; Van der Loos and Woolsey, 1973) or enucleation disrupts retinotopic map and interneuron migration in the LG (Golding et al., 2014; Wiesel and Hubel, 1963). Such effects, however, are less visible in HO nuclei, consistent with their circuit position, which is more remote from the periphery (Frangeul et al., 2014, 2016; Theyel et al., 2009).

In molecular terms, input ablation at early postnatal ages in mice leads to an HO-like nucleus identity in FO nuclei, both in terms of molecular identity and connectivity (Frangeul et al., 2016; Grant et al., 2016). Likewise, genes normally enriched in LG during development are preferentially downregulated following monocular enucleation (Giasafaki et al., 2022). Although these results support the idea that HO-type genetic identity is a developmental ground-state feature of thalamic neurons and the FO identity is acquired secondarily in an input-dependent manner, this has not been directly tested and is the topic of the present study.

Here, using single-cell and single-nucleus RNA sequencing of the developing mouse thalamus between E11.5 and E18.5, we first identify the timeline of the emergence of FO and HO neuron molecular identity and, within these classes, that of LG, LP, VB and Po identities, and identify transcriptional programs that are conserved and distinct between these cells. We show that HO identity emerges earlier than FO identity, and that enucleation prevents the normal maturation of LG neurons in a cell type-specific manner, while LP neurons are only minimally affected. Our results establish a mechanistic framework for the diversification of thalamic neuron types during development, in which HO nuclei start differentiating earlier than FO nuclei, with peripheral input playing a cell type-specific role in FO neuron differentiation.

## RESULTS

### Identification of the developmental transcriptional programs of thalamic nuclei

Essentially all mouse thalamic neurons are born between embryonic day (E) 10.5 and E13.5 (see https://neurobirth.org/ and Fig. S1) (Baumann et al., 2023 preprint). As a first foray into the developmental molecular diversity of FO and HO nuclei, we performed single-cell RNA sequencing of prosomere 2 (p2), from which the thalamus arises, at E11.5, E12.5, E13.5, E14.5, E16.5 and E18.5. In select experiments, we performed FlashTag labeling (Govindan et al., 2018) to label structure boundaries – in particular, the zona limitans intrathalamica – allowing precise microdissection (Fig. 1B). Both the pTH-R and pTH-C progenitor domains were collected, which will giving rise to GABAergic and glutamatergic neurons, respectively (Vue et al., 2007, 2009). After tissue collection and dissociation, 10X single-cell RNA-sequencing, quality control, data filtering, and bioinformatic removal of GABAergic cells (see Materials and Methods and Figs S2 and S3), we obtained a total of 18,977 transcriptionally characterized cells, which we displayed in two dimensions using UMAP dimensionality reduction (Fig. 1C-E). This approach revealed that cells were molecularly organized based on their type and differentiation state. Progenitors grouped together and were the predominant cell type until E13.5, when neurogenesis is largely complete, while neurons progressively emerged throughout time, reflecting progressive maturation of age-specific cohorts (Fig. 1C). This developmental timeline was confirmed using markers of progenitors and postmitotic neurons, respectively (Fig. 1D). Unbiased cluster analysis revealed a total of 19 clusters corresponding to four classes of cells (Fig. 1E): apical progenitors, expressing *Nes* and *Sox2*; basal progenitors, which expressed *Neurog2* and *Btg2* (Moreau et al., 2021; Telley et al., 2016); and early and late neurons, which expressed classical markers of neuronal differentiation (*Tubb3* and *Id4*; Menezes and Luskin, 1994; Yun et al., 2004) and maturity [*Eno2* and *NeuN* (*Rbfox3*); Duan et al., 2016; Schmechel et al., 1978], respectively (Table S1, Fig. S4). Within progenitors, two types of cells were present, i.e. apical progenitors and basal progenitors (Table S2, Fig. S5) (Wang et al., 2011). Analysis of differentially expressed genes across clusters and cell types unveiled the sequential functional transcriptional programs at play in the developing thalamus: initially, genes involved in regulation of cell division and proliferation predominated, with the presence of transcripts such as *Cdca8* and *Cenpf*, respectively, while axon- and synapses-related genes were expressed in early and late neurons with transcripts such as *Epha3* and *Dlg2*, respectively (Fig. 1F, Tables S1, S3). Finally, the specificity of the genes identified was confirmed using the Allen Developing Mouse Brain Atlas *in situ* database (Allen Institute for Brain Science, 2004; https://developingmouse.brain-map.org). AP- and BP-specific genes were enriched in the germinal zones of the developing thalamus (in the ventricular and subventricular zones, respectively). EN- and LN-specific genes were expressed in the mantle zone of prosomere 2, at early and late developmental stages, respectively (Fig. 1G, Fig. S4). Together, these data identify the core molecular cell types present in the developing thalamus.

**Fig. 2.**
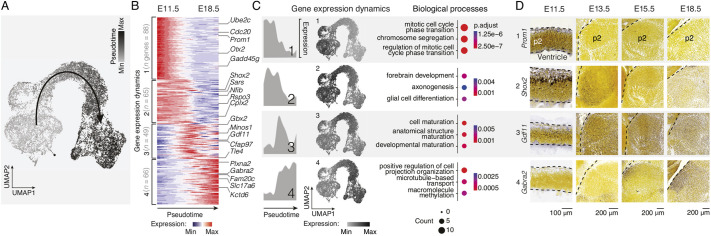
**Core thalamic developmental programs are sequentially expressed in four molecular waves.** (A) Pseudotime values on the UMAP embedding showing the developmental continuum-like organization of cells. (B) Unbiased clustering of genes based on expression dynamics reveals four distinct transcriptional waves sequentially expressed along the pseudotime axis. Examples of selected differentially expressed genes are provided for each wave (left). (C) The four distinct transcriptional waves reveal sequential expression peaks (left). Average of expression of each wave shows a discrete pattern of expression along the UMAP representation following the pseudotime axis (middle). An example of biological processes of gene ontologies associated with each transcriptional wave (right). (D) *In situ* hybridization sections of selected differentially expressed genes showing distinctive expression in their respective wave. Image credit: Allen Institute for Brain Science (https://developingmouse.brain-map.org/). Scale bars: 100 μm in D (E11.5); 200 μm in D (E13.5, E15.5 and E18.5).

To get a finer-grained understanding of the sequential molecular programs that are unfolding in thalamic cells during this developmental period, we used a pseudo-differentiation approach (here called ‘pseudotime’, for simplicity). Across this axis, we identified transcriptional waves of sequential gene expression, i.e. genes that were most dynamically expressed across time (Fig. 2A, Fig. S6). This approach revealed four main waves of transcription for a total of 268 genes that progressed in function from control of cell cycle properties, followed by axonogenesis, cellular maturation and microtubule organization (Fig. 2B,C, Tables S4 and S5). Here, again, validation using available *in situ* datasets for select genes confirmed the temporal progression observed with the single-cell analysis (Fig. 2D). Comparison of this dataset with a corresponding dataset obtained in the cortex (Telley et al., 2019) revealed differentially expressed genes and ontologies (Table S6). For example, hedgehog signaling pathway transcripts are present early in differentiating thalamic cells, while Hippo signaling pathway transcripts are enriched in the early developing cortex. Both pathways are major regulators of cell proliferation and differentiation (Komada, 2012; Lin et al., 2012; Zhong et al., 2024), suggesting that different strategies are at play to give rise to cellular diversity within the progenitors of these two structures. Together, these data identify the temporal transcriptional programs at play in the developing thalamus.

### Emergence of FO and HO neuron identity

**Fig. 3.**
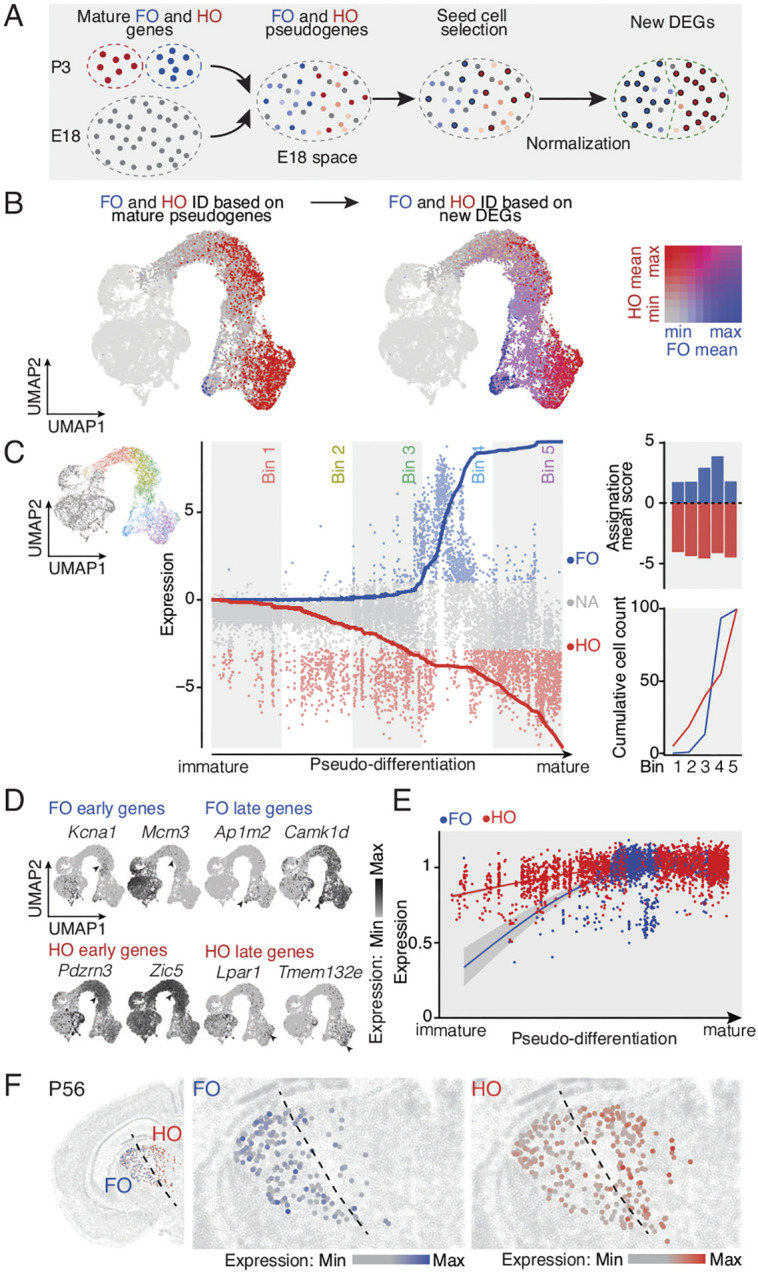
**Emergence of thalamic first and high order identities occurs at distinct paces.** (A) Schematic illustration of the strategy used for first order (FO) and higher order (HO) final cell selection during embryonic development. (B) UMAP representation of FO and HO mature pseudogenes (left, 15 FO genes, 15 HO genes from previously published data; Frangeul et al., 2016, see Table S7) allowing the prediction of FO and HO differentially expressed genes during embryonic development (right). (C) Each post-mitotic cell is assigned with its FO/HO ratio score and the cumulative cell count is represented (center). Assignation mean score of the cells into five consecutives bins and normalized cumulative cell count plot show an early emergence of HO identity compared with FO (right). (D) Example feature plot of select early and late FO/HO genes. (E) Average temporal dynamic and expression levels of FO and HO shared genes on FO and HO cells. (F) Spatial transcriptomic analysis of select differentially expressed genes in FO and HO thalamic nuclei (FO, *n*=15 genes; HO, *n*=23 genes available in spatial transcriptomic dataset from Zhang et al., 2023). FO, first order; HO, higher order.

We next examined how FO and HO molecular identity emerged during development. Previous work has identified several gene markers that distinguish these two types of nuclei in the early postnatal brain and adulthood (Frangeul et al., 2016) (Table S7), yet when and how these identities emerge remains unclear. A limiting factor to bioinformatically fate-map FO- and HO-type neurons based on adult markers is the fact that they are not necessarily expressed at earlier stages of development. To circumvent this limitation, we designed an approach in which we ranked our most mature E18.5 cells based on their relative expression of previously identified P3 FO and HO marker genes, and defined the top-ranked cells as ‘seed cells’. Using these seed cells only, we identified new differentially expressed genes to iteratively serve as markers for analysis in earlier developmental ages (Fig. 3A). Cells were then defined as FO- or HO-type based on above-threshold expression of the resulting new gene set, with level of expression of the E18 newly identified genes corresponding to the level of confidence in FO or HO assignment (Fig. 3B and Table S8). Publicly available *in situ* hybridizations (Allen Institute for Brain Science, 2004; Fig. S8A) validated this approach by revealing a corresponding distribution the newly identified FO and HO markers. This analysis revealed that HO identity emerges earlier and over a more prolonged period than FO identity, the latter being restricted to a transient period at the end of the pseudo-differentiation window (Fig. 3C and Fig. S7).

We then identified differentially expressed genes in FO- and HO-type cells during development. This revealed a prolonged early wave of gene expression in HO neurons, consisting of around 1350 early and 700 late genes, while FO neurons instead progressed rapidly to more mature states with around 550 early and 1300 late genes (Fig. 3D, Fig. S8 and Table S9). When genes expressed both by FO and HO were examined, their time course was more transient in FO, suggesting different paces of maturation (Fig. 3E and Table S8). Genes expressed specifically by FO neurons included *Slc6a4*, *Camk4* and *Rps29*, which are involved in synaptic vesicle cycle, long-term potentiation and ribosomal biogenesis, respectively. Genes expressed specifically by HO-type cells included *Robo1*, *Alcam* and *Grin2a*, which are involved in biological processes such as axon guidance, cell adhesion and synaptic transmission, respectively (FO, *n*=861 genes; HO, *n*=723 genes; Fig. S9, Tables S8 and S11).

Gene ontology network analysis highlighted that FO and HO mostly share common molecular pathways, suggesting that these thalamic regions have a common origin (Fig. S9 and Table S11). The specificity of the genes identified was confirmed using an available spatial transcriptomic dataset (Zhang et al., 2023): FO-specific genes were enriched in FO nuclei of the thalamus, while HO-specific genes were mostly expressed in HO nuclei of the thalamus, albeit with less specificity than FO genes, perhaps reflecting more generic features of HO transcriptional programs (Fig. 3F). Analysis using a recently published dataset of thalamic nucleus-specific genes (Govek et al., 2022) confirmed these findings: when order-specific genes from this dataset were used as input in our labelling strategy, order-specific label assignments and expression scores were similar to those obtained above (Fig. S8C and Table S10). Accordingly, 38.5% of our top 100 FO and 100 HO markers were present in this dataset. We determined the timing of divergence between FO and HO neurons by analyzing gene expression at single serial timepoints between E13 and E18. FO- and HO-type cells clustered together until E14 and became clearly distinct by E16 (Fig. S8D). This coincides with hindbrain sensory axons reaching putative FO nuclei (Pouchelon et al., 2012), suggesting that input may influence the emergence of FO neuron identity. Together, these data reveal that HO molecular identity emerges mostly before FO molecular identity, and identify the transcriptional programs at play during the development of these two types of thalamic nuclei.

### Emergence of VB, LG, Po and LP neuron identity

**Fig. 4.**
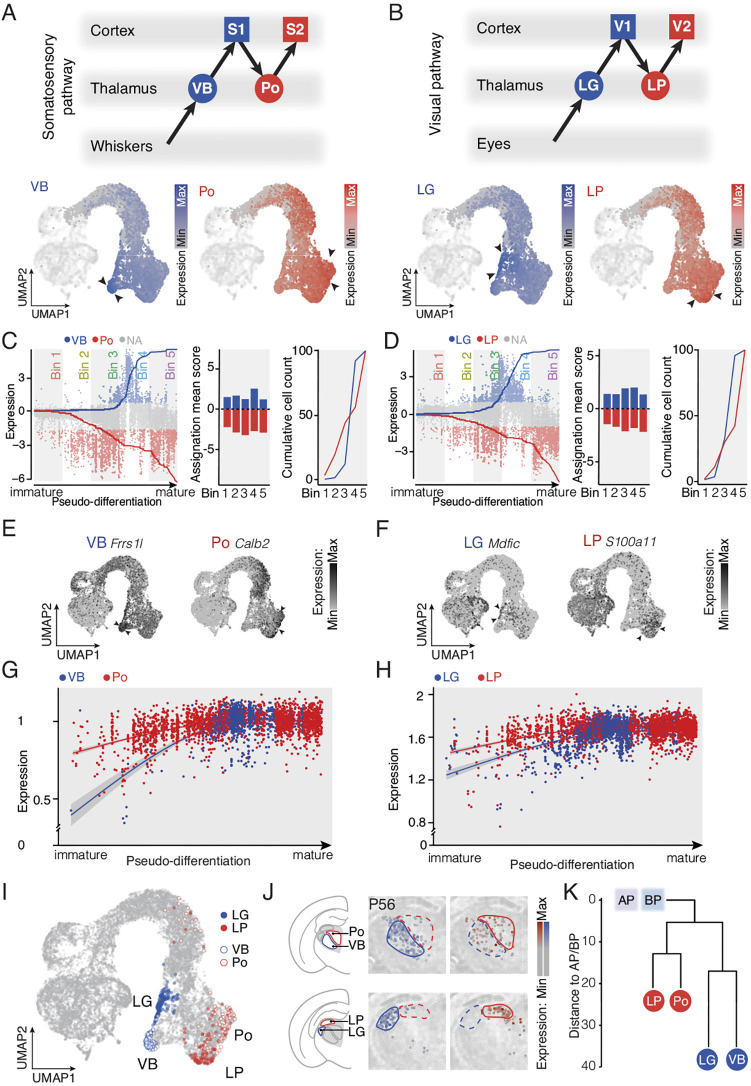
**Acquisition of nuclei-specific identities during development relies on both shared and distinct molecular programs.** (A,B) Schematic representation of a somatosensory pathway (A, top) and visual pathway (B, top). UMAP representation of VB and Po (A), and LG and LP (B) differentially expressed genes during embryonic development using the same strategy as in Fig. 3B. (A, bottom) 10 VB genes and 10 Po genes, and (B, bottom) 10 LG genes and 10 LP genes from previously published data (Frangeul et al., 2016, see Table S12). (C,D) Each post-mitotic cell is assigned with its VB/Po (C) or LG/LP (D) ratio score and the cumulative cell count is represented (left). Assignation mean score of the cells into five consecutive bins and normalized cumulative cell count plot show early emergence of Po identity compared with VB (C, right) and an early emergence of LP identity compared with LG (D, right). (E,F) Example feature plots of select genes for VB and Po (E), and LG and LP (F). (G) Average temporal dynamic and expression levels of VB and Po (G), and LG and LP (H) shared genes on VB and Po, and LG and LP cells, respectively. (I) UMAP representation of VB, Po, LG and LP cells for late timepoints after the selection process. (J) Spatial transcriptome of select differentially expressed genes showing distinctive expression in VB, Po, LG and LP thalamic nuclei (LG, *n*=15 genes; LP, *n*=11 genes; VB, *n*=11 genes; Po, *n*=16 genes available in spatial transcriptomic dataset from Zhang et al., 2023). (K) Hierarchical clustering depicting the LG, LP, VB and Po organization, and distances to the AP and BP cell categories. LG, dorsolateral geniculate nucleus; LP, pulvinar/latero-posterior nucleus; Po, posteromedial nucleus; S1, primary somatosensory cortex; S2, secondary somatosensory cortex; V1, primary visual cortex; V2, secondary visual cortex; VB, ventrobasal nucleus.

Building on the strategy above, we next examined the emergence of modality-specific FO and HO nuclei, i.e. the VB and Po for the somatosensory system and the LG and LP for the visual system. Using the seed-cell approach, but this time with VB- and Po-specific genes (Fig. 4A), and LG- and LP-specific genes (Fig. 4B, Tables S12 and S13), we examined the time course of emergence of neurons with these identities (Fig. 4C,D and Fig. S10) and corresponding dynamic gene expression programs (Fig. 4E,F and Table S14).

Based on the analyses above, the final identity of these four nuclei could be established, allowing definition of nucleus-specific markers (Fig. 4I and Table S13). VB/Po (Fig. S11A) and LG/LP (Fig. S11B) shared common molecular pathways with specific genes involved in their respective FO and HO nuclei. Gene ontology networks were strongly shared across modalities (57.5% of shared gene ontology across modalities, Table S15), confirming conserved developmental programs across modalities consistent with previous results (Frangeul et al., 2016). When genes expressed both by VB and Po or LG and LP were examined, their time course was more transient in FO nuclei, suggesting different paces of maturation (Fig. 4G,H). An available spatial transcriptomic dataset (Zhang et al., 2023) revealed that these newly identified genes had a spatial distribution appropriate for their identity (Fig. 4I,J). Hierarchical clustering of cells with VB, Po, LG and LP identities revealed stronger similarities within hierarchical levels (i.e. VB and LG versus Po and LP, Fig. 4K) than within modalities (i.e. VB and Po versus LG and LP), consistent with previous results in the mature thalamus (Frangeul et al., 2016). Moreover, LP and Po are molecularly closer to progenitors than VB and LG are, supporting the hypothesis that HO nuclei molecular identity appears before FO molecular identity (Fig. 4K). Together, these data reveal that LP molecular identity emerges before LG molecular identity, and Po emerges before VB, by identifying new molecular markers for these four thalamic nuclei during development.

### Input-dependent differentiation of the LG is neuron-type-specific

**Fig. 5.**
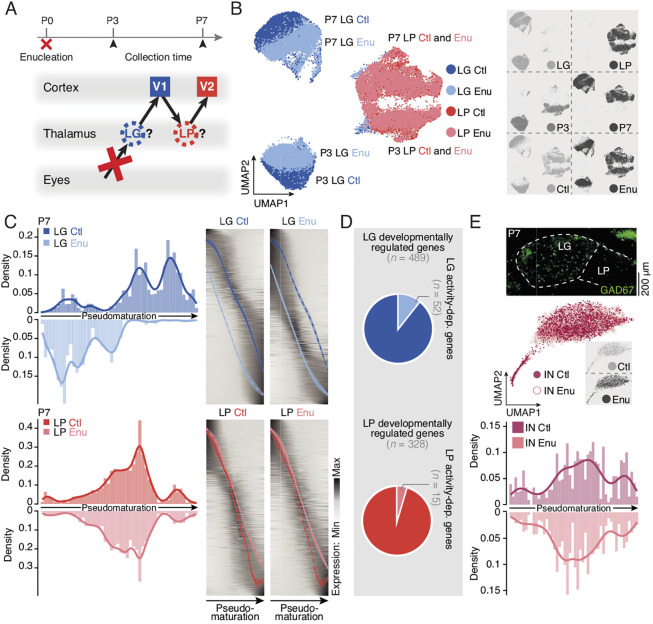
**Cell type-specific transcriptional responses to input deprivation.** (A) Schematic illustration of the experimental timeline and the procedure for microdissection. (B) UMAP representation of the single-cell RNA sequencing dataset color coded by condition (LG Ctl, dark blue; LG Enu, light blue; LP Ctl, dark red; LP Enu, light red). Insets are UMAP representations of the different conditions LG and LP (top right), P3 and P7 (middle right), and Ctl and Enu (bottom right). (C) Each LG Ctl and LG Enu (top left), and LP Ctl and LP Enu (bottom left) cell is placed along the pseudotime axis. Specific transcriptional waves for LG Ctl and LG Enu (top right), and LP Ctl and LP Enu (bottom right) lineages are represented. (D) Pie chart representing the number of LG (top, *n*=52) or LP (bottom, *n*=15) activity-dependent genes among developmentally regulated genes (LG, *n*=489; LP, *n*=328). (E) Coronal section in GAD67^GFP^ mice showing interneurons (INs) in the LG (top). UMAP representations of the IN in Ctl and Enu conditions [middle; insets are a UMAP representation of the different conditions (Ctl and Enu)]. The density of each IN Ctl and IN Enu cell is plotted along the pseudotime axis (bottom). Ctl, control; Enu, Enucleation; IN, interneuron; LG, dorsolateral geniculate nucleus; LP, pulvinar/latero-posterior nucleus; V1, primary visual cortex; V2, secondary visual cortex. Scale bar: 200 μm.

Focusing on visual pathways, we examined the extent to which the developmental transcriptional programs of the LP and LG are driven by input-dependent processes after birth. For this purpose, we performed bilateral enucleation at P0, collected LG and LP at P3 and P7, and, using single-cell RNA sequencing, assessed and compared molecular identities across ages and conditions (Fig. 5A and Fig. S12).

In glutamatergic neurons (Fig. 5B), LG identity evolved between P3 and P7, while LP neuron identity was more stable, as indicated by progression in PCA distances (Fig. S13A). After enucleation, P7 glutamatergic LG neuron identity was strikingly affected, while LP neuron identity remained stable (Fig. 5B, Fig. S13A). Accordingly, the number of differentially expressed genes affected after input deprivation was higher in glutamatergic LG neurons than in LP neurons (P7 LG Enu decrease, *n*=93; P7 LG Enu increase, *n*=57; P7 LP Enu decrease, *n*=23; P7 LP Enu increase, *n*=22; Fig. S13B, Table S16), confirming and extending previous results (Frangeul et al., 2016).

Analysis of differentially expressed genes revealed a downregulation of *Dcc*, a netrin receptor, in the LG after enucleation, while another axon guidance molecule, *Robo2*, was upregulated. Interestingly, Dcc and Netrin signaling, and Robo and Slit signaling have distinct roles in axonal guidance, which could provide the basis for the distinct target specificities of FO and HO axons (Deiner et al., 1997; Lo Giudice et al., 2019; Thompson et al., 2009; Zhang et al., 2012). Downregulated genes in the LG after enucleation were involved in cholinergic synapse and calcium signaling, while upregulated genes were involved in GABAergic synapse and glutamatergic synapse function (Fig. S13C,D and Table S17). In LP neurons, *Lrrc4c*, a netrin G1 ligand, was downregulated after enucleation, while *Il1rapl2*, a member of the interleukin 1 receptor family playing a role at synapses, was upregulated. Owing to the low number of differentially expressed genes in the LP between conditions, the only relevant ontologies were modulation of synaptic transmission and homo/heterophilic cell adhesion via plasma membrane adhesion molecules (Fig. S13, Tables S16 and S17). Although acquisition of LP identity in LG Enu neurons was not visible using PCA distance-based analysis (Fig. S13A), targeted analysis using only the LP marker genes identified above (Fig. 4I and Table S13) showed that 16/57 LG Enu upregulated genes were also LP markers (Fig. S14C). This suggests some level of acquisition of LP molecular identity in LG Enu neurons, as previously shown (Frangeul et al., 2016).

We next examined how input deprivation affects the temporal unfolding of transcriptional programs in LG and LP neurons using the pseudotime alignment approach previously applied. This revealed that input-deprived LG neurons lagged in their differentiation compared with control LG neurons, highlighting a crucial role for retino-thalamic input in driving the maturation of glutamatergic neurons in this nucleus (Fig. 5C). In the LG, 10.6% of developmentally regulated genes were affected by input deprivation, whereas this was the case for only 4.6% of developmentally regulated LP genes (Fig. 5D). Unbiased clustering identified molecularly distinct clusters of LG glutamatergic neurons (Fig. S15A,B and Table S18). Post-enucleation, the maturation of clusters 0 and 3 appeared particularly affected (Fig. S15C). Differential gene expression analysis did not provide immediate clues as to why these clusters may be more sensitive (Table S19); e.g. cluster 0 neurons expressed *Gabrr1* and *Hcn1* (involved in ion transport), and cluster 3 expressed *Kcns3* and *Scnn1a* (involved in ion transport), and *Trabd2b*, *Wnt6* and *Shisa6* (involved in Wnt signaling) (Table S18). The overlapping spatial distribution of these clusters suggests that their topology is not a factor determining their sensitivity (Fig. S15D). Hence, one may speculate that differences in innervation by hindbrain versus cortical afferents could play a role in this process. Finally, in contrast to the input-dependent sensitivity of glutamatergic neuron differentiation, when inhibitory interneurons were examined, the temporal unfolding of their transcriptional programs was unaffected (Fig. 5E), revealing that, even within a single nucleus, cell type-specific input-dependent processes are at play.

## DISCUSSION

Our findings reveal that HO neuron identity is a developmental ground state, while FO neuron identity emerges later. Upon input deprivation in the visual system, the differentiation of FO glutamatergic neurons is delayed, while HO identity is not detectably affected. Similarly, inhibitory neurons are not detectably affected. Thus, even within single thalamic nuclei, neuronal types have distinct susceptibilities to input-dependent differentiation.

A fundamental difference between FO and HO inputs is the topographical organization of their synaptic inputs. Hence, whereas the PrV nucleus of the trigeminal nerve projects in a somatotopic manner to the VB, projections to the Po are, instead, less precise; a corresponding difference exists for projections of VB and Po nuclei to their cortical targets (Herkenham, 1980; Pouchelon et al., 2012). Similarly, HO-type projections in the cortex appear to rely mostly on metabotropic synaptic transmission, while ionotropic channels mediate FO transmission, arguably allowing a more precise temporal coding of information. These observations may account for the fact that FO nuclei (here, the LG) are more sensitive than HO nuclei (here, the LP) to input deprivation.

The observation that interneurons are not detectably transcriptionally affected by input deprivation has not previously been reported and may reflect the fact that this cell type invades the LG relatively late, during the first few postnatal days. We have, however, previously shown that enucleation affects the migration of these cells within this nucleus, as well as their synaptic integration within visual thalamic circuits (Golding et al., 2014), such that these effects may be occurring at the post-transcriptional level or rely on extranuclear transcription, as they are not detected here.

Our results strongly suggest that subsets of HO neurons are developmentally co-opted from FO neurons in an input-dependent manner; this is also suggested by previous work in which FO neurons do not acquire FO identity and instead have a sustained HO-like identity (Frangeul et al., 2016; Grant et al., 2016). In principle, FO neurons could represent an distinct population of neuron that mature late, but there is no evidence in birthdating studies for this: FO and HO nuclei are born simultaneously (see https://neurobirth.org/) (Baumann et al., 2023 preprint). Similarly, in principle, FO neurons could be born at the same time but migrate into the thalamus – the structure we are collecting – at later stages, giving a sampling-related illusion of late differentiation. Here, again, there is no evidence of such a migration occurring. Instead, the most parsimonious explanation is that FO neurons emerge from subsets of HO neurons to acquire new properties in an input-dependent manner. HO neurons are still detected after the emergence of FO neurons, the latter being a transient event, suggesting that only subsets of HO neurons become FO neurons.

From an evolutionary standpoint, our findings thus support the idea that FO-type neurons could have developed from subsets of ancestral HO-type neurons. FO neurons might have been evolutionarily co-opted from some HO neuron types based on higher ability to transmit signals from high-resolution body receptors, perhaps reflecting their distinct metabolic, electrophysiological and connectivity characteristics (Wong-Riley, 1979; Wong-Riley and Welt, 1980; Yamashita et al., 2013). Performing enucleations at an earlier timepoint *in utero* (Moreno-Juan et al., 2017) could help study the role of input in identity acquisition, as the postnatal enucleations performed here are carried out at a time when some level of retinal innervation is already present. It will thus be interesting in future studies to assess precisely how input-dependent processes regulate the gene regulatory networks at play in these cell-type-specific fate decisions.

## MATERIALS AND METHODS

### The use of generative AI and AI-assisted technologies in the writing process

The authors used Chat-GPT to assist them with the phrasing of some sections of the text, which were then modified further. They take full responsibility for text content.

### Mouse strains

The experiments were carried out in compliance with Swiss laws and obtained approval from both the Geneva Cantonal Veterinary Authorities and their ethics committee. The study strictly adhered to the ARRIVE guidelines. CD1 male and female mice were sourced from Charles River Laboratory. For embryonic experiments, mice were mated within an early morning 3 h window at the Charles River facilities. In-house matings were conducted overnight for post-natal experiments, with the following morning designated as E0.5. Before the experiment, all mice were housed at the institutional animal facilities under animal caretaker control for their veterinary needs, covering the alternation of light and dark cycles, a 22°C temperature, and unlimited water and food provided. Transgenic mice consist of GAD67^GFP^ knock-in mice (Golding et al., 2014; Tamamaki et al., 2003).

### Surgical procedures

#### FlashTag *in utero* injection

FlashTag *in utero* injections were carried out as previously described (Govindan et al., 2018). Pregnant mice were anesthetized by isoflurane at gestational time point E11, positioned on a 37°C heated surgical platform for the duration of the surgeries. During surgeries, small incisions were made in the abdominal region to reveal the uterine horns, and 1 μl of 10 mM FlashTag (carboxyfluorescein succinimidyl ester, CellTrace CFSE, Life Technologies, C34554) was intracerebroventricularly injected by a nanoinjector equipped with pulled, beveled and dust-cleaned glass pipet. After the procedure, the uterine horns were carefully repositioned within the abdominal cavity, and sutures were used to close the peritoneum and skin. The mice were then placed on a warming pad until full recovery from anesthesia.

#### Bilateral enucleation

Bilateral enucleation surgeries were carried out on P0 pups as previously described (Golding et al., 2014). A small incision was made in the eyelid with a scalpel, and the eye was separated from the optic nerve with scissors and removed from the orbit with forceps. Pups were placed in a box on a heating pad for recovery before returning to their mothers.

### scRNA-seq collection and sequencing

Single-cell library captures were performed on 6-12 pooled embryos, depending on the time point, with one library for each condition. The samples were composed of dissociated cells and nuclei from the thalamic space of E11.5, E12.5, E13.5, E14.5, E16.5 and E18.5 embryos (Fig. 1B) for embryonic experiments, and of independent dissections of LG and LP in both control and enucleation conditions for P3 and P7 time points (Fig. 5).

#### Cell dissociation and FAC-sorting

Diencephalons of the embryos were collected in ice-cold Hank's Balanced Salt Solution (HBSS) and segments were microdissected under a stereomicroscope. Thalamic chunks were then incubated in 200 µl single-cell dissociation solution consisting of papain (1 mg/ml)-enriched HBSS at 37°C for 10 min with trituration every 2 min. Cells were further dissociated via gentle up-and-down pipetting and the reaction was stopped with the addition of 400 µl of 2 mg/ml ovalbumin-enriched ice-cold FACS buffer [2 mg/ml glucose, 0.1% BSA, 1:50 citrate phosphate dextrose from (Sigma, C7165), 10 U/ml DNase I and 1 μM MgCl_2_]. The cell suspension was then passed through a 40 µm cell strainer to remove cellular aggregates. Cells were then centrifuged for 5 min at 500 ***g*** at 4°C. After removal of the liquid, the pellet was suspended in 250 µl of ice-cold FACS buffer and the resulting solution was finally sorted on a MoFloAstrios device (Beckman) to reach a concentration of 410 cells/µl.

#### Nuclei isolation

Diencephalons of the embryos were collected in ice-cold Hank's Balanced Salt Solution (HBSS) and segments were microdissected under a stereomicroscope. Thalamic chunks were frozen and stored at −80°C until further processing.

Nuclei were isolated using an EZ Nuclei isolation kit as previously described (Santinha et al., 2023). In brief, the tissue was resuspended in 2 ml of ice-cold extraction buffer (Nuclei EZ prep, Sigma NUC101), gently dounce-homogenized (KIMBLE Dounce tissue grinder, Sigma D8938, six strokes with A and B pestle) and incubated for 5 min in a total of 4 ml EZ buffer. The extracted nuclei were collected by centrifugation (500 ***g*** at 4°C for 5 min) and supernatant was removed followed by resuspension and incubation in EZ buffer for 5 min. After centrifugation, the nuclear pellet was washed in 4 ml washing buffer containing 1% BSA in PBS, with 50 U/ml of SUPERase-In (Thermo Fisher, AM2696) and 50 U/ml of RNasin (Promega, N2611). The nuclei were resuspended in 1 ml of washing buffer, filtered through a 30 μm strainer, stained with Hoechst (Invitrogen H3570, 1:500) for 5-10 min and processed for fluorescence-activated nuclei sorting on a Beckman Coulter MoFlo Astrios. 20,000 nuclei were sorted and 42 µl of nuclei suspension was used to load the 10X Genomics snRNA-seq preparation.

#### 10X single cell RNA sequencing

Cell suspensions were captured using 10X Genomics Chromium Single Cell 3′ v3 reagents following the manufacturer's protocol. The quality control of the cDNA and libraries was performed using Agilent's 2100 Bioanalyzer. Subsequently, the libraries were sequenced using the HiSeq 4000 sequencer. The resulting FASTQ files obtained from the sequencing were processed and mapped with 10X Genomics Cell Ranger pipeline using the GRCm38 mouse genome as a reference. Default parameters for read mapping, counting and quality controls were used as described in the Cell Ranger V6.0.0 documentation.

#### C1 single cell RNA sequencing

Cells stained with FlashTag were captured using the C1-Fluidigm system. Following the previously mentioned dissociation protocol, 8 µl of cell suspension was sorted on a MoFloAstrios to enrich for the top 20% most FlashTag-positive cells. Each sample representing a different embryonic time points was mixed with C1 suspension reagents (2 µl; Fluidigm) for a total cell suspension volume of ∼10 µl, containing ∼500 cells per µl. This mixture was then loaded onto the C1 Single-Cell AutoPrep integrated fluidic circuit (HT-800, Fluidigm, 100-57-80). cDNA synthesis and pre-amplifications were conducted following the manufacturer's instructions for the C1 system (Fluidigm). Captured cells were imaged using the ImageXpress Micro Widefield High Content Screening System (Molecular Devices). Single-cell RNA-sequencing libraries of the cDNA were prepared using the Nextera XT DNA library prep kit (Illumina). Libraries were multiplexed and sequenced in accordance with the manufacturer's recommendations, employing paired-end reads on the HiSeq4000 platform (Illumina).

### Single cell RNA-seq data analysis

All single cell transcriptomics analyses were performed using R Statistical Software (v4.2.2), the Seurat V4 package and Bioconductor packages (Hao et al., 2021; Huber et al., 2015; https://www.r-project.org/). Graphs and visualization were generated with the ggplot2 package (Wickham).

For quality control, independent libraries were processed as Seurat objects. All cells with fewer than 200 detected genes and genes expressed in fewer than three cells were removed. In detail, for the FlashTag condition, we integrated 614 FlashTag cells at E11.5, 310 at E12.5, 303 at E13.5, 241 at E14.5 and 93 at E18.5, and, for non-FlashTag nuclei, 2066 at E11.5, 2449 at E12.5, 2658 at E13.5, 3013 at E14.5, 3652 at E16.5 and 3578 at E18.5 to create the embryonic UMAP embedding (Fig. 1C). Concerning postnatal experiments, we retained 4950 cells for the control LG P3 condition, 2679 for the control P3 LP, 5543 for the enucleation P3 LG, 3551 for the enucleation P3 LP, 5445 for the control P7 LG, 3483 for the control P7 LP, 6043 for the enucleation P7 LG and 5109 for the enucleation P7 LP. PCA was performed on variable genes to reduce dimensionality of the dataset. UMAP was based on the reduced dimensional space of the 20 most significant dimensions of the PCA using the UMAP package Barnes-Hut version of Seurat with a perplexity set at 30 (Fig. 1C). A UMAP-based clustering analysis was then performed by the shared nearest neighbor (SNN) modularity optimization algorithm (Waltman and van Eck, 2013). The number of independent genes detected ranged between 2684 and 3994, with an average of 3148 by cluster for the embryonic dataset, and between 762 and 7630, with an average of 2451 by cluster for the postnatal dataset. Differentially expressed genes between clusters were obtained by Seurat-implemented Wilcoxon rank sum tests with default parameters. The cluster identities in this UMAP space were uncovered by feature plots of typical cell type marker genes (Fig. 1E,F). The first three embedded dimensions of the UMAP were output for further use to represent the pattern of various features during differentiation. The minimal distance parameter of the UMAP was set to 0.3 and the number of neighboring points used in local approximations was set to 30.

The doublet scores computation was performed by the recent computeDoubletDensity method of the scDblFinder R package (V 1.18.0). All scores were normalized between 0 and 100 to represent the probability of being a doublet. The timing of divergence between FO and HO was determined by isolating time points and representing the average expression of the top 15 FO and HO pseudogenes. Each time point and representation were processed as previously described for normalization and dimensionality reduction.

#### Merging of datasets

Individual datasets were integrated together following the scRNA-seq integration vignette of Seurat documentation with following steps. Individual experiments were normalized by the NormalizeData Seurat function with default parameters, and 2000 variable features were computed with the ‘vst’ selection method. Integration anchors were then computed for 30 dimensions with a k-filter parameter of 50, and used for the final integration. Integration of embryonic and postnatal datasets was not performed because of the large maturity difference between P7 and embryonic cells. This maturity gap leads to both contracted and sparse pseudo-differentiation values, reducing the temporal resolution of our main period of interest.

#### Pseudotime projection

Pseudo-differentiation and pseudotime scores were assigned using the monocle 3 R library following the published pipeline, with default parameters (Fig. 2A, Fig. S6). Pseudo-time analysis was performed with Monocle 3 using genes that have passed the quality control of the Seurat object creation (Qiu et al., 2017). Genes considered as defining the progression of the pseudo-time were those that were detected as having an expression above 0.5 by Monocle 3. Negative binomial was considered for the model encoding the distribution that describes all genes. During the pseudo-time processing, the dimensionality of the dataset was reduced by the Discriminative Dimensionality Reduction with Trees (DDRTree) algorithm on the log-normalized dataset with ten dimensions considered.

#### Transcriptional waves

Transcriptional wave analysis was performed with default parameters as described previously (Telley et al., 2016) (Fig. 2B) and the bmrm V4.1 R package. Briefly, genes presenting large variations were regrouped along a pseudo-time axis, forming clusters composed of similar time-dependent gene expressions. These clusters of patterns were labeled as waves and processed for further GO term enrichment (Fig. 2C).

GO term, pathways enrichment analysis and their representations were performed using the R pathfindR package (Ulgen et al., 2019), in accordance with the library documentation and DAVID bioinformatics resources 6.8 (Huang et al., 2009), on a list of differentially expressed genes obtained by *findmarker* and *findallmarker* Seurat functions (Figs 1F and 2C). Gene ontology networks were a follow-up analysis of the Seurat lists of differentially expressed genes. After the list was created using the *findmarker* Seurat function with default parameters, the gene names, log-fold changes and adjusted *P*-values were inputted into the run_pathfindR function of the pathfinder R package (v 2.2.0), with the following parameters: custom_genes defined as mmu_kegg_genes and custom_descriptions=mmu_kegg_descriptions, as described in the mice-specific PathfindR workflow (https://cran.r-project.org/web/packages/pathfindR/vignettes/non_hs_analysis.html). The combine_pathfindR_results function was then used to compare results of different conditions and obtain specific and common pathways (Figs S9B, S11A,B and S13C-E).

#### Spatial transcriptomics and label transfer

A coronal representation of a whole-brain spatial transcriptomics experiment dataset was obtained (Zhang et al., 2023) and imported into R. Coronal level 37 was selected as it is representative of the studied thalamic space (Figs 3F, 4J and Fig. S15D). Spatially computed cells were processed as previously described, with steps including normalization and dimensionality reduction, followed by clustering and marker genes analysis to retain only glutamatergic cells. Thalamic nuclei of interest were manually defined based on anatomical markers (Figs 3F and 4I). We generated pseudogenes based on the order- and nuclei-specific marker genes, filtered by a log-fold-change threshold (logFC>1), as identified in our embryonic analysis (Tables S8 and S13). Spatial expression was confirmed by taking advantage of the Allen Brain Atlas ISH dataset (Allen Institute for Brain Science, 2004) FO, *n*=4 genes; HO, *n*=4 genes; VB, *n*=4 genes; Po, *n*=6 genes; LG, *n*=6 genes; LP, *n*=4 genes; Figs S8A and S11A,B). The LG P7 control and deprivation clusters, as previously described (Fig. 5B), were integrated after the merging of datasets procedure outlined in the previous paragraph. The control condition served as the reference, and its subclustering labels (Seurat clusters 0 to 7) were transferred to the deprivation dataset (Fig. S15A). Gene markers of these control sub-clusters that were common to expressed genes in the spatial dataset (all genes specific to each sub-cluster with a positive log fold-change after the *findallmarker* Seurat function) were used to compute a pseudogene for each subcluster based on their mean expression. These pseudogenes were then represented for both the control and deprivation conditions (Fig. S15A). Subsequently, the representative expressions of these sub-clusters were individually plotted along the pseudotime values of each cell (Fig. S15C) and on the spatial dataset (Fig. S15D).

#### *In situ* hybridization

High resolution *in situ* hybridization images were selected and extracted from the Allen Brain Atlas (Allen Institute for Brain Science, 2004) using R scripts to connect and download from the Allen Brain Atlas Application Programming Interface (http://mouse.brain-map.org/static/api) (Figs 1G; 2D). Regarding order and nucleus-specific markers, we selected available genes from the top 100 most expressed markers genes in each category (Figs S8A and S11A,B). Out of eight FO/HO mature markers present in the Allen Brain ISH Atlas (Doc2b, Gbx2, Hcrtr1, Htr2c and Plxnc1 for HO, and Gpr88, Spp1 and Tgfb3 for FO), five were still expressed at P14 and only two at P56, consistent with a maturation-related expression.

#### Detection of embryonic FO, HO, LG, LP, VB and Po cells based on mature markers

We selected the top 10 most differentially expressed genes (based on fold-change value) for FO, HO, LG, VB, Po and LP by relying on a previously published study reporting gene expression at P3 (Frangeul et al., 2016) (Tables S7 and S12). A pseudogene was generated in each condition by averaging the expression of the categorical markers (i.e. hierarchy or nucleus markers) across all cells of our dataset; E18 cells in the top 99th quantile of pseudogene expression were defined as ‘seed cells’ (Fig. 3A). New differentially expressed genes were identified based on these seed cells, using the FindAllMarkers Seurat function; the top 15 most differentially expressed genes (based on fold-change value) were used for the generation of a second round of pseudogenes. Cells were ascribed a FO or HO identity (respectively VB or Po, or LG or LP), based on which pseudogene had the highest level of normalized expression, ultimately retaining only cells within the top 70th quantile of absolute expression values (FO-HO – VB-Po and LG-LP, respectively) (Figs 3C and 4C,D).

#### Validation of cell identities using an external dataset (Govek et al., 2022)

We extracted the top 10 markers genes from Govek et al. (2022) using VPM, VPL/VMG and DLG/VMG/VPL gene expression lists for FO neurons, and PO, DMG, LD, CM and MD gene expression lists for HO neurons. These 10 genes were used to compute pseudogene expression in each condition, as described above (Fig. S8C, left), yielding similar identity assignment scores (Fig. S8C, right).

## Supplementary Material

10.1242/develop.202764_sup1 Supplementary information

Table S1. Apical progenitors, basal progenitors, early neurons and late neurons differentially expressed genes list (related to Fig. 1E).

Table S2. Apical and basal cell cycle progenitors differentially expressed genes list (related to Fig. S5).

Table S3. Apical progenitors, basal progenitors, early neurons and late neurons gene ontology analysis (related to Fig. 1F).

Table S4. Gene expression across transcriptional waves along pseudotime (related to Fig. 2B).

Table S5. Gene ontologies associated with transcriptional waves (related to Fig. 2C).

Table S6. Comparison of developmental thalamic and cortical waves (related to Fig. 2B,C).

Table S7. List of mature FO and HO genes used to define new differentially expressed genes (related to Fig. 3B).

Table S8. FO and HO new differentially expressed genes and shared genes list (related to Fig. 3B).

Table S9. FO and HO waves gene list (related to Fig. 3D).

Table S10. List of FO and HO genes from Govek et al., 2022 (related to Fig. S8C).

Table S11. Biological processes associated with FO and HO transcriptional maps clusters (related to Fig. S9).

Table S12. List of mature VB, Po, LG and LP genes used to define new differentially expressed genes (related to Fig. 4A and B).

Table S13. VB, Po, LG and LP new differentially expressed genes and shared genes list (related to Fig. 4A, B, G, H and I).

Table S14. VB, Po, LG and LP waves gene list.

Table S15. Biological processes associated with VB, Po, LG and LP transcriptional maps clusters (related to Fig. S11).

Table S16. LG and LP Ctl vs. Enu differentially expressed genes list (related to Fig. 5B, S13 and S14).

Table S17. Biological processes associated with LG Ctl, LG Enu, LP Ctl and LP Enu transcriptional maps clusters (related to Fig. S13).

Table S18. P7 LG control subclusters marker genes and associated biological processes (related to Fig. S15).

Table S19. P7 LG subclusters Ctl. Vs. Enu. differentially expressed genes list and associated biological processes (related to Fig. S15).
