# Supplementary material for: Developmental emergence of first- and higher-order thalamic neuron molecular identities
Source: Development. 2024 Sep 30;151(18):dev202764. doi: 10.1242/dev.202764 (PMC11463969; doi:10.1242/dev.202764)
Supplement: Supplementary information [file develop-151-202764-s1.pdf]

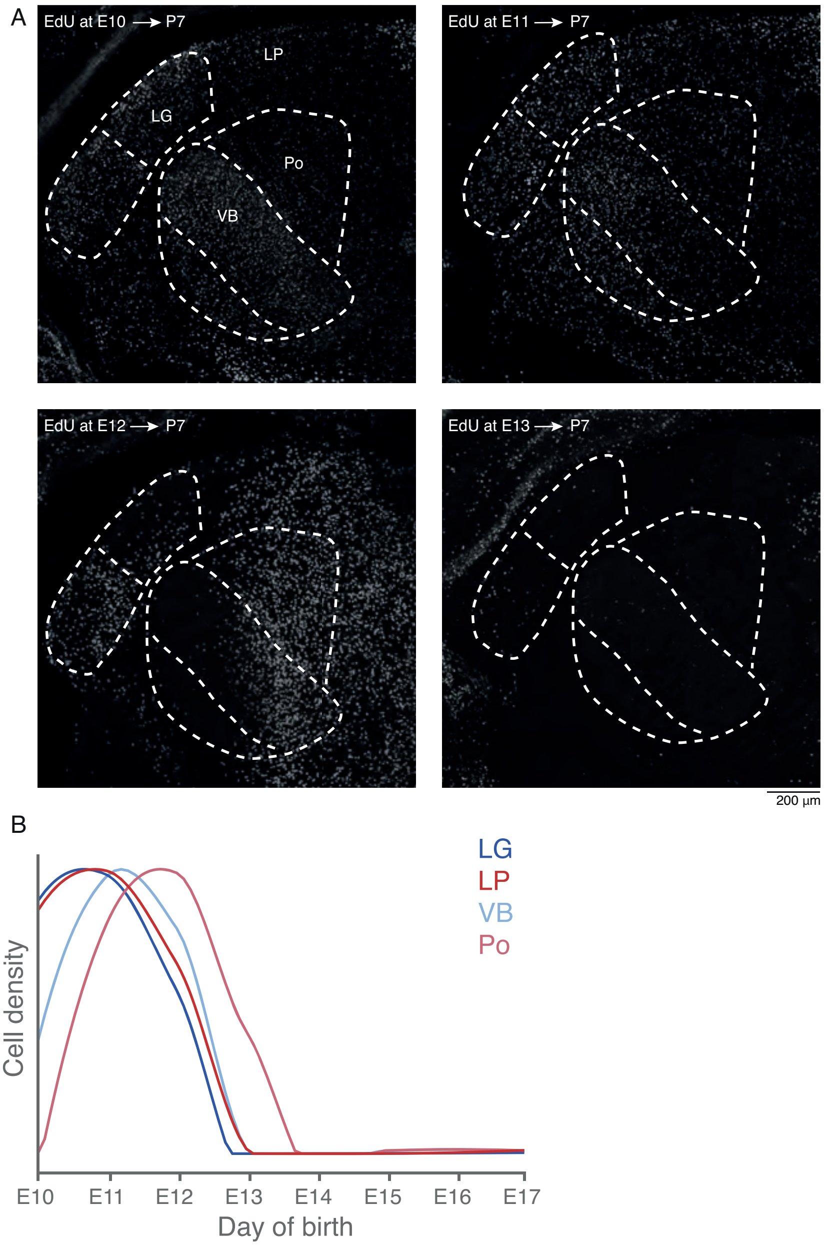

Fig. S1. Final thalamic localization of VZ- and SVZ-born neurons between E10 and E13. (A) Example images showing thalamic nuclei of P7 mice injected with EdU at different embryonic stages. (B) Line plot displaying the birth date of VB, Po, LG and LP thalamic nuclei (data from https://neurobirth.org/) (Baumann et al., 2023). LG, dorsolateral geniculate nucleus; LP, pulvinar/latero-posterior nucleus; Po, posteromedial nucleus; B, ventrobasal nucleus. Scale bar: 200 µm (A).

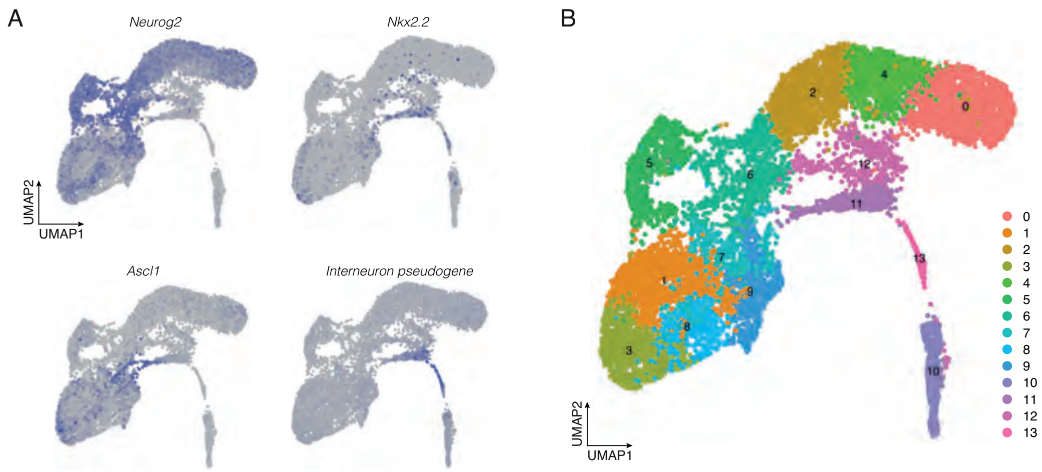

Fig. S2. Identification of thalamic GABAergic clusters. (A) Feature plot of *Neurog2* (marker of pTH-C which gives rise to all thalamic nuclei), *Nkx2.2* and *Ascl1* (marker of pTH-R which gives rise to the GABAergic neurons of the thalamus) expression. The Interneuron pseudogene UMAP represent the mean expression of 8 interneurons markers: *Gad1*, *Gad2*, *Sst*, *Gabbr1*, *Gabbr2*, *Reln*, *Nr2f2* and *Nkx2.2*. (B) Initial UMAP showing the different clusters. Clusters 11 and 13 representing the GABAergic neurons lineage were removed for this study.

Development • Supplementary information

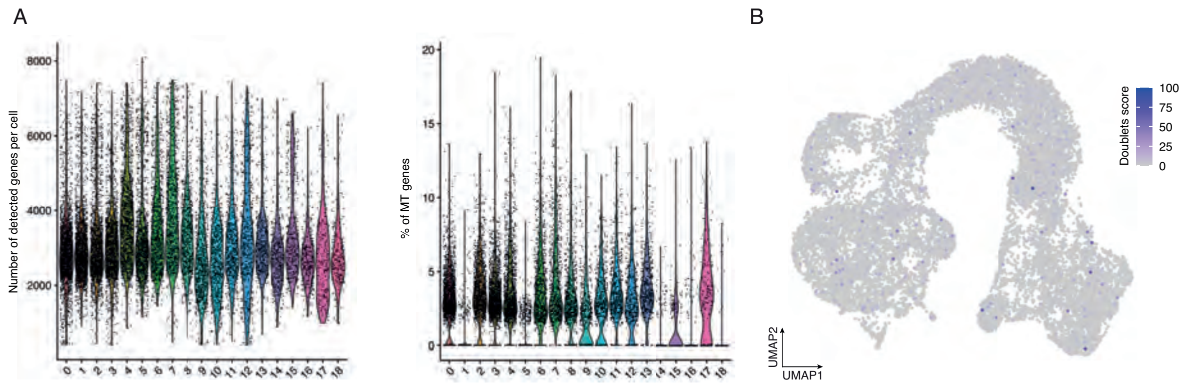

Fig. S3. Quality control for prenatal scRNA-seq datasets. (A) Violin plot illustrating the number of detected genes by cells and clusters, along with percentage of mitochondrial genes (% MT). (B) UMAP showing doublet scores for each cell from the scDblFinder R package. Colored cells were moved in front of the representation to prevent overlapping effects. Given the low number of potential doublets, those cells were not removed in the analysis.

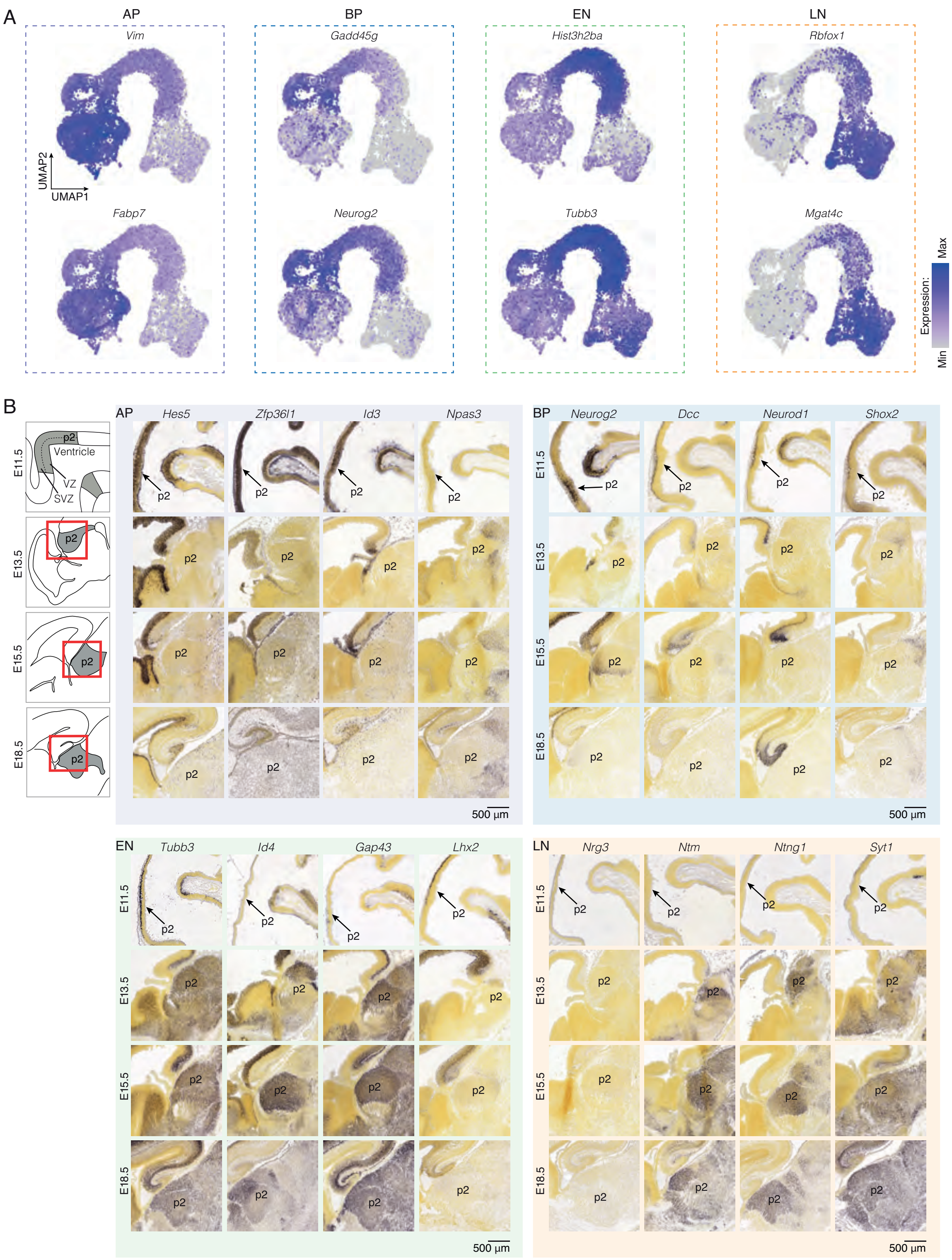

Fig. S4. Expression of select genes in AP, BP, EN and LN. (A) Example feature plot of select genes for apical progenitors (AP), basal progenitors (BP), early neurons (EN) and late neurons (LN). (B) In situ hybridization (ISH) sections of selected differentially expressed genes showing distinctive expression during mouse embryonic development; image source: Allen Developing Mouse Brain Atlas (developingmouse.brain-map.org). Scale bar: 00 µm (B).

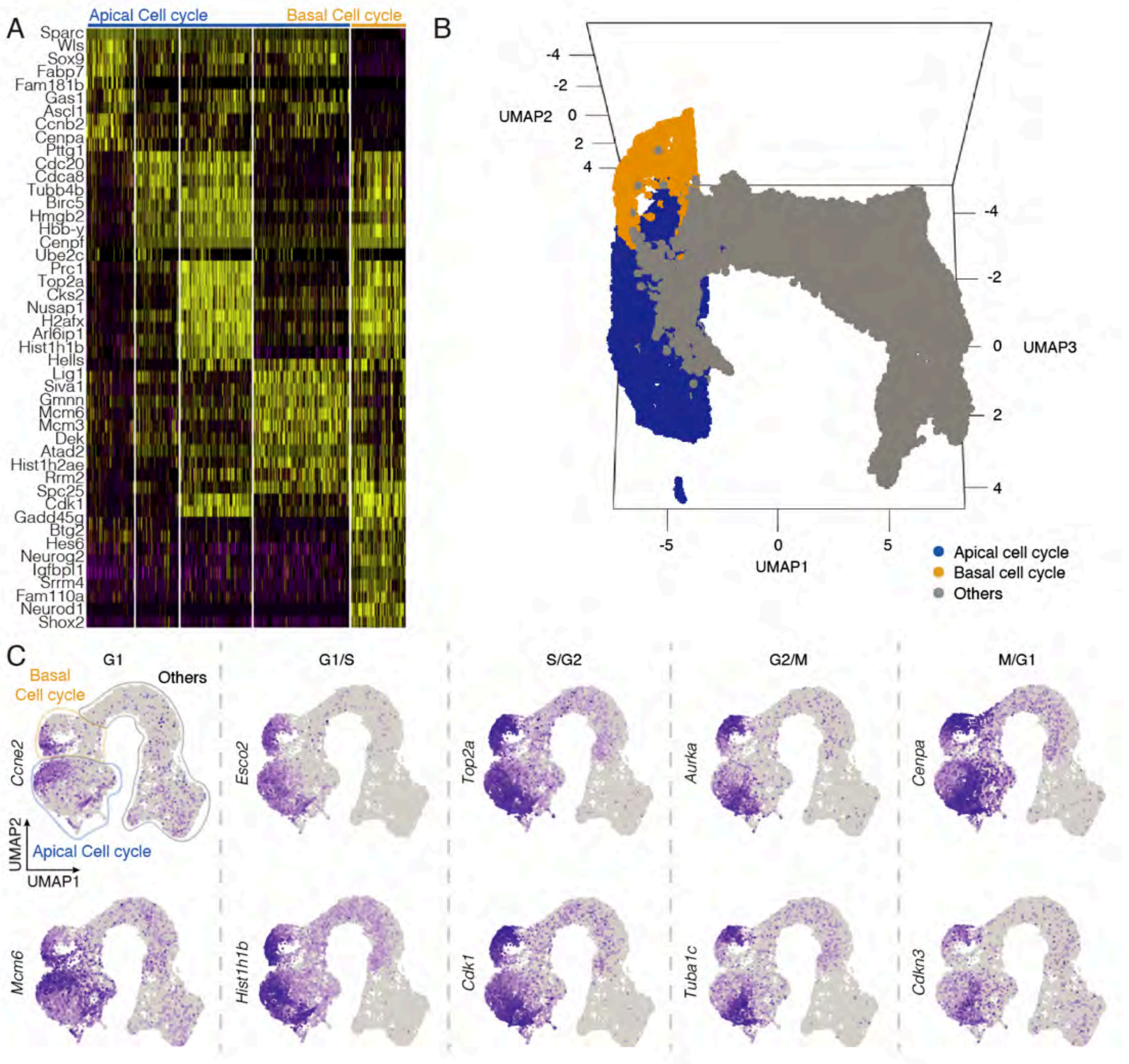

Fig. S5. Molecular signature of apical and basal cell cycle in the developing thalamus. (A) Expression of the top 10 of each cell cycle phase highlight the presence of apical and basal cell cycle. (B) UMAP representation in 3D showing the apical and basal cell cycle. (C) Example feature plot of selected genes for each cell cycle phase.

Development • Supplementary information

A

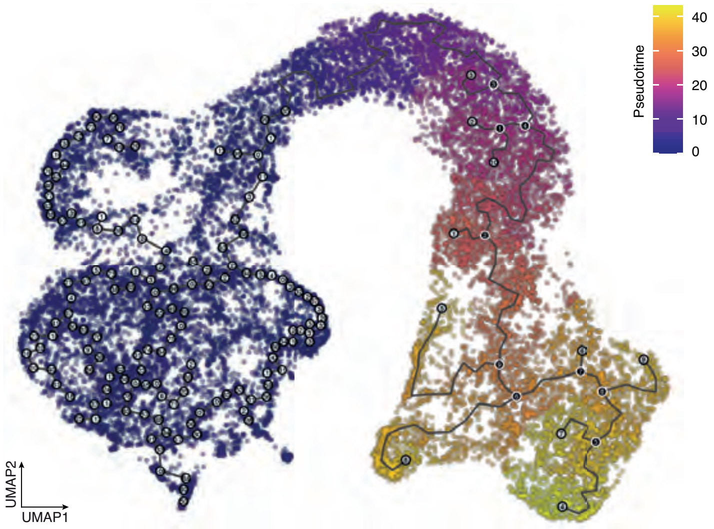

Fig. S6. Pseudotime organisation of the embryonic dataset. (A) Pseudotime color-coded UMAP representation of the embryonic space with Monocle3 trajectories tree.

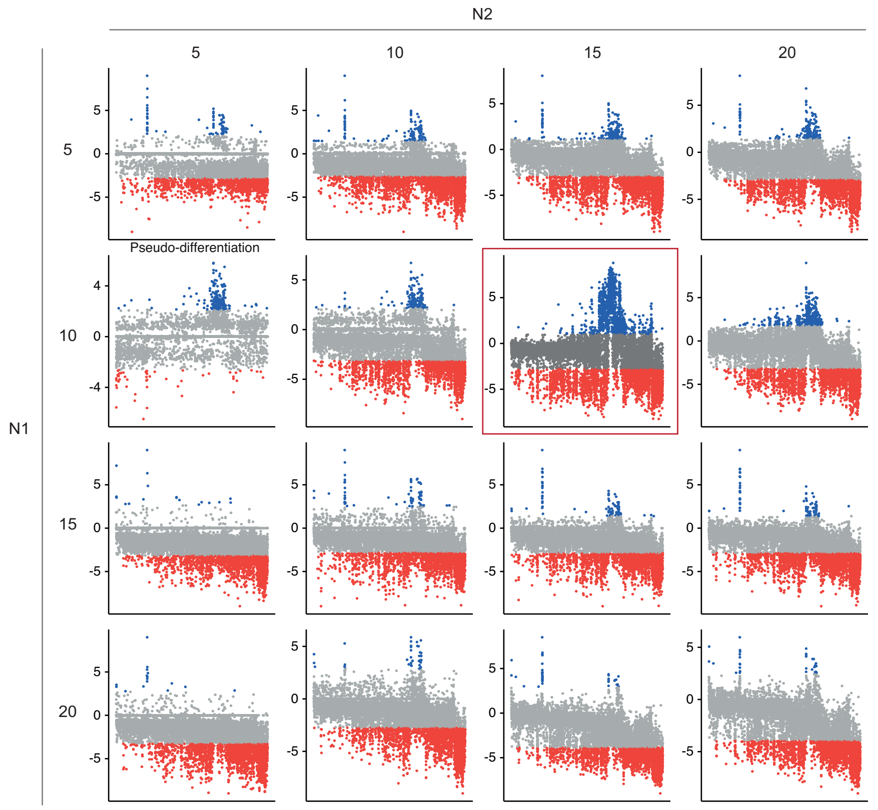

Fig. S7. Benchmarking of number of genes used for FO/HO marker selection. 2D array representing couples of selected numbers of genes for the FO/HO assignation method and their output. The final selected parameters (N1 = 10 and N2 = 15) is highlighted in red.

Development • Supplementary information

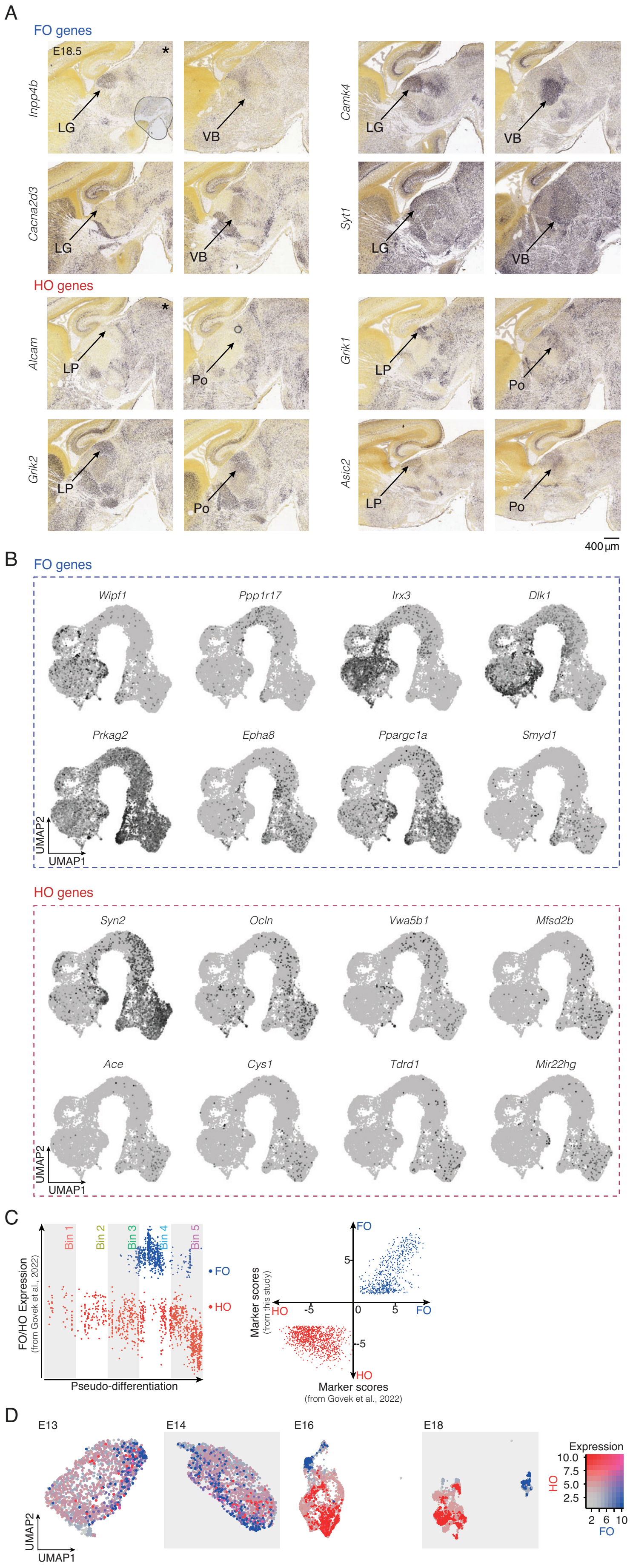

Development • Supplementary information

Fig. S8. Expression of select genes in FO and HO thalamic neurons. (A) In situ hybridization (ISH) sections of selected FO and HO differentially expressed genes at E18.5; image source: Allen Developing Mouse Brain Atlas (developingmouse.brain-map.org). The left images for *Inpp4b* and *Alcam* (highlighted with an asterisk) also appear in Fig. S11B to illustrate LG and LP identity, respectively. (B) Example feature plot of select FO and HO wave genes. (C) Comparison with external dataset from Govek et al., 2022. Top 10 markers genes for FO and HO nuclei were selected and used as seed on E18 cells to test FO/HO selection pipeline. Left: The resulting graph shows similar selection and pseudo-differentiation dynamics as ours. Right: Scatter plot of FO and HO scores resulting of the selection methods are compared between this study and markers from Govek et al., 2022. X-axis: this study scores. Y-axis: Govek et al., 2022. (D) Expressions of top FO and HO marker pseudogenes across independent time points reveal a mixed identity at E13 and E14, which becomes segregated by E16. FO, first order; HO, higher order. Scale bar: 400 µm (A).

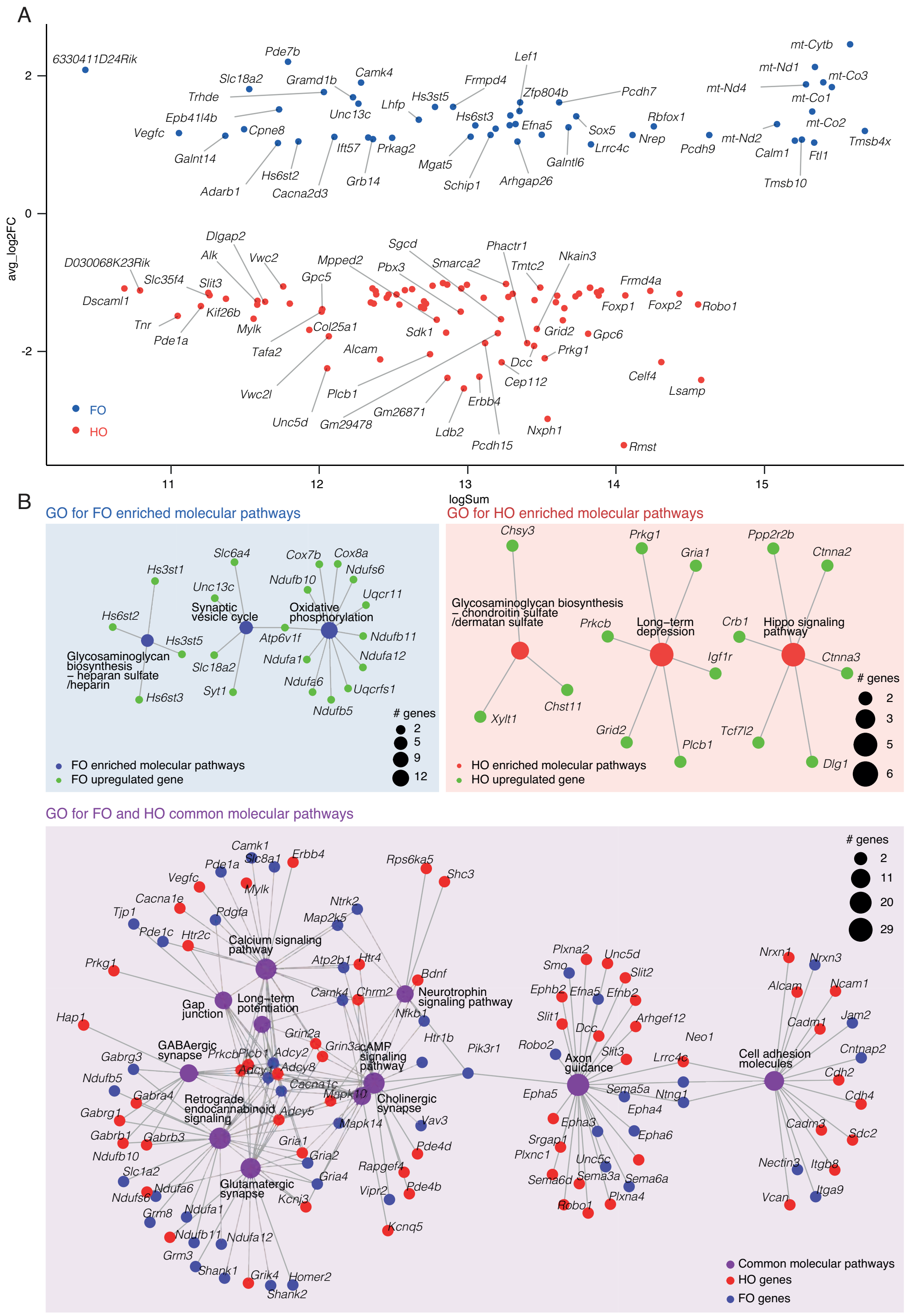

Fig. S9. Molecular signature and gene ontology of FO and HO thalamic neurons. (A) MA plot showing log fold-change (y-axis) compared with mean expression value (x-axis) (log Fold-Change >1 for FO, <(-1) for HO, logSum > 8 and p-value < 0.01), negative fold change representing specific genes in HO (red) and positive fold change representing specific genes in FO (blue). (B) String plots representing gene ontology networks for FO enriched molecular pathways (blue), HO enriched molecular pathways (red) and FO and HO common molecular pathways (purple). FO, first order; HO, higher order.

Development • Supplementary information

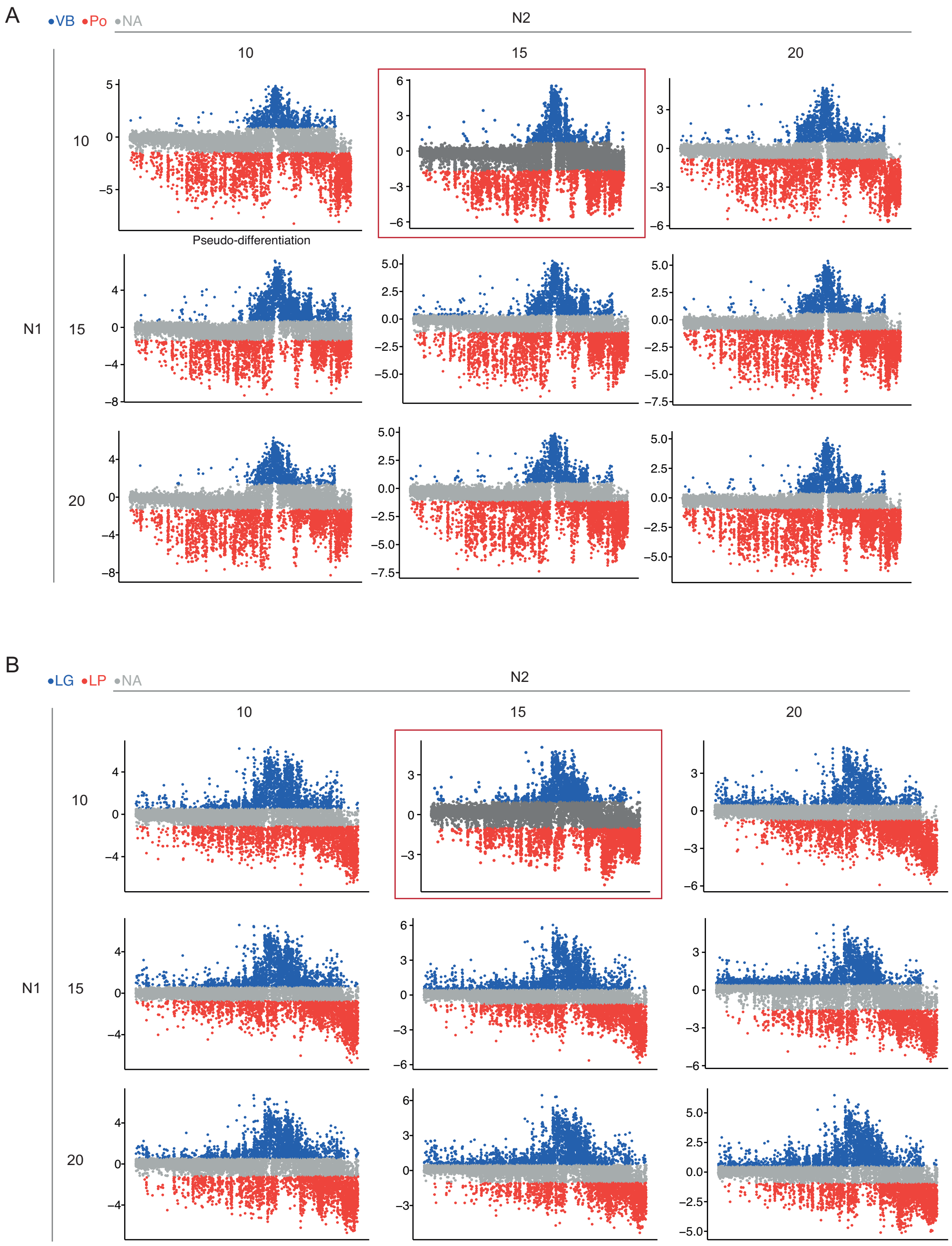

Fig. S10. Benchmarking of number of genes used for nuclei marker selection. (A) 2D array representing couples of selected numbers of genes for VB/Po assignation method and their output. The final selected parameters (N1 = 10 and N2 = 15) is highlighted in red. (B) 2D array representing couples of selected numbers of genes for LG/LP assignation method and their output. The final selected parameters (N1 = 10 and N2 =15) is highlighted in red.

Development • Supplementary information

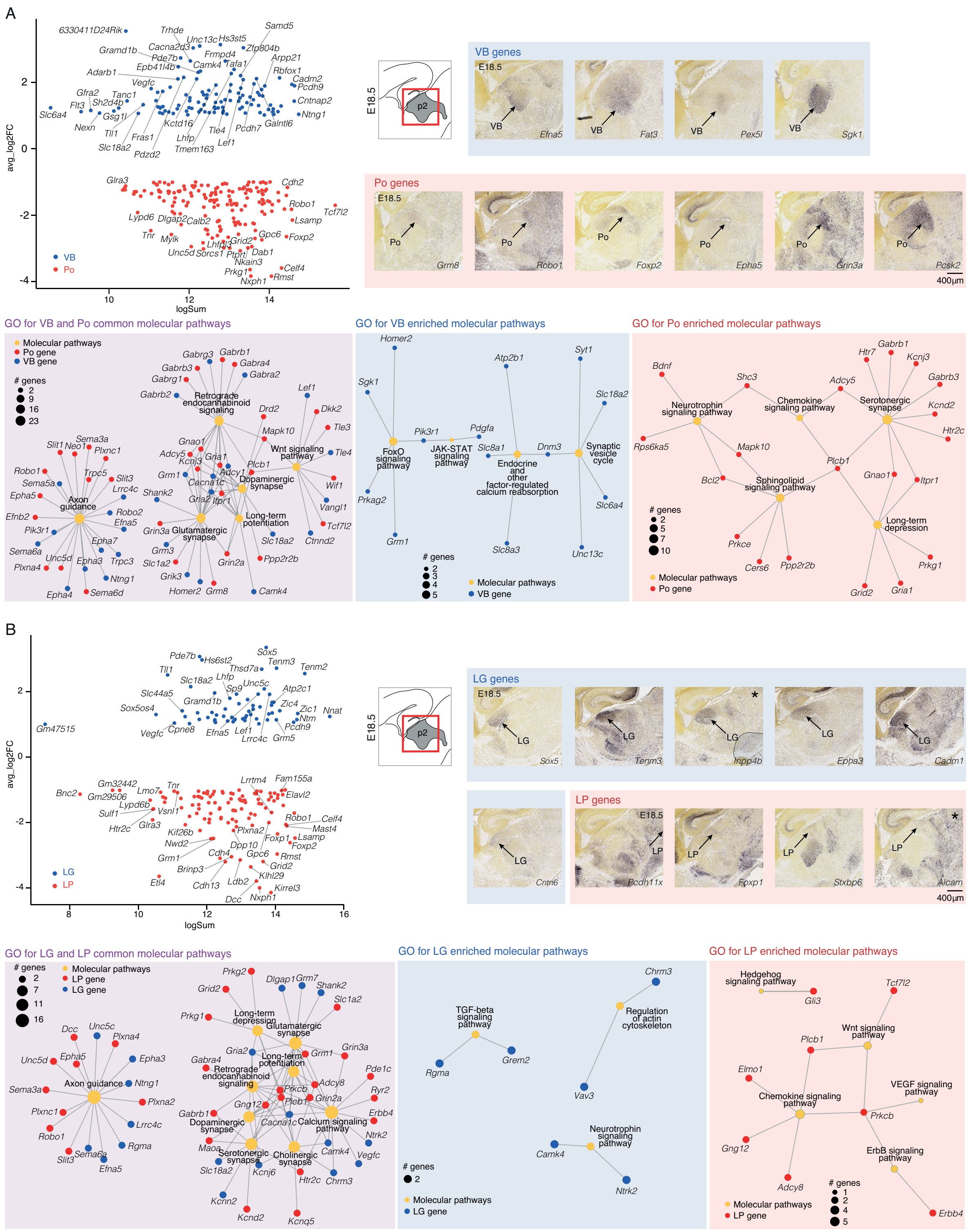

Fig. S11. Molecular signature and gene ontology of nucleus-specific thalamic neurons. (A) MA plot showing log fold-change (y-axis) compared with mean expression value (x-axis) (same criteria as in Fig. S9), negative fold change representing specific genes in Po (red) and positive fold change representing specific genes in VB (blue) (top left). In situ hybridization (ISH) sections of selected VB and Po differentially expressed genes at E18.5; image source: Allen Developing Mouse Brain Atlas (developingmouse.brain-map.org) (top right). String plots representing gene ontology networks for VB enriched molecular pathways (blue), Po enriched molecular

pathways (red) and VB and Po common molecular pathways (purple) (bottom). (B) MA plot showing log fold-change (y-axis) compared with mean expression value (x-axis), negative fold change representing specific genes in LP (red) and positive fold change representing specific genes in LG (blue) (top left). In situ hybridization (ISH) sections of selected LG and LP differentially expressed genes at E18.5; image source: Allen Developing Mouse Brain Atlas (developingmouse.brain-map.org) (top right). The third image illustrating LG genes (*Inpp4b*) and the fourth image illustrating LP genes (*Alcam*) (highlighted with an asterisk) also appear in Fig. S8A to illustrate FO and HO identity, respectively. String plots representing gene ontology networks for LG enriched molecular pathways (blue), LP enriched molecular pathways (red) and LG and LP common molecular pathways (purple) (bottom). LG, dorsolateral geniculate nucleus; LP, pulvinar/latero-posterior nucleus; Po, posteromedial nucleus; VB, ventrobasal nucleus. Scale bar: 400 µm (A,B).

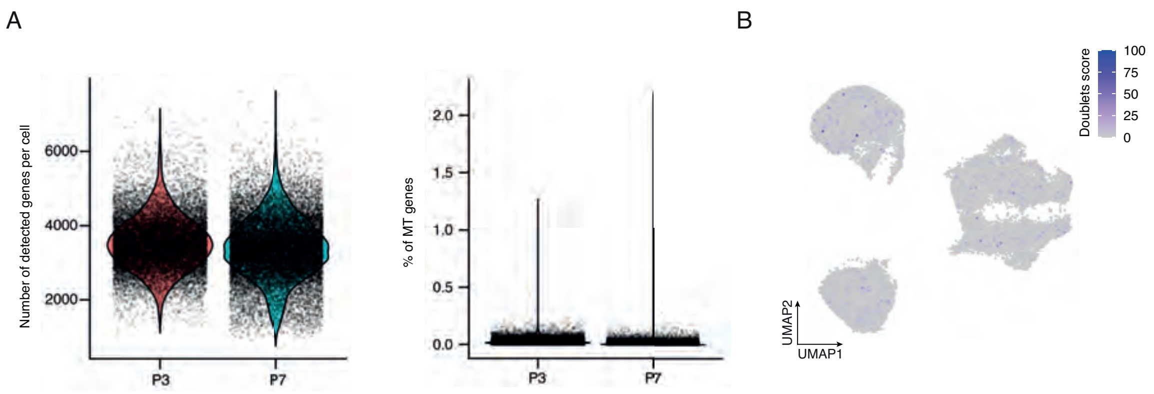

Fig. S12. Quality control for postnatal scRNA-seq datasets. (A) Violin plot illustrating the number of detected genes by cells and time-points along with percentage of mitochondrial genes (% MT). (B) UMAP showing doublet scores for each cell from the scDblFinder R package. Colored cells were moved in front of the representation to prevent overlapping effects. Given the low number of potential doublets, those cells were not removed in the analysis.

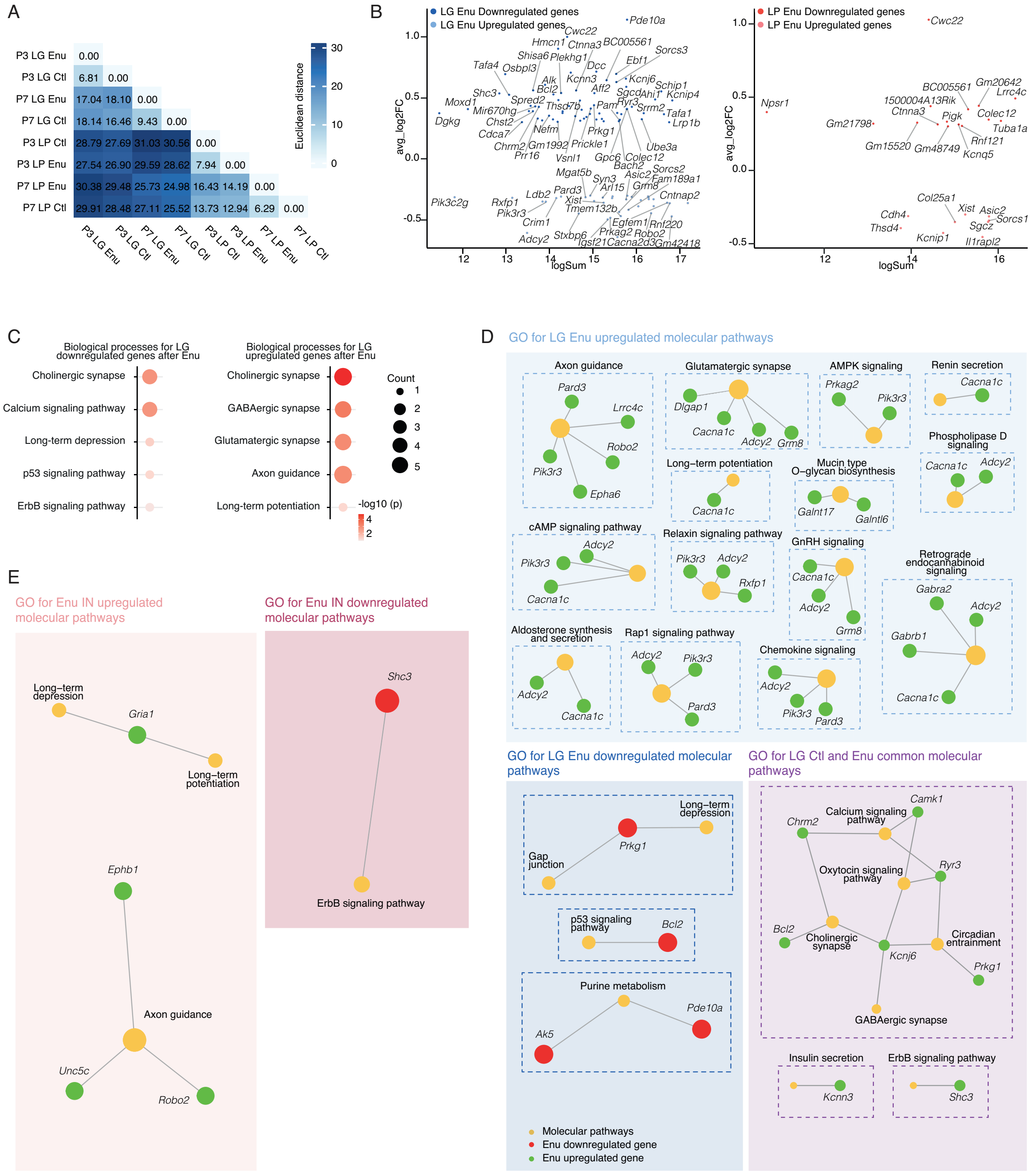
A
P3 LG Enu 0.00
P3 LG Ctl 6.81 0.00
P7 LG Enu 17.04 18.10 0.00
P7 LG Ctl 18.14 16.46 9.43 0.00
P3 LP Ctl 28.79 27.69 31.03 30.56 0.00
P3 LP Enu 27.54 26.90 29.59 28.62 7.94 0.00
P7 LP Enu 30.38 29.48 25.73 24.98 16.43 14.19 0.00
P7 LP Ctl 29.91 28.48 27.11 25.52 13.73 12.94 6.29 0.00
P3 LG Enu
P3 LG Ctl
P7 LG Enu
P7 LG Ctl
P3 LP Ctl
P3 LP Enu
P7 LP Enu
P7 LP Ctl
Euclidean distance
30
20
10
0
B
LG Enu Downregulated genes
LG Enu Upregulated genes
avg_log2FC
logSum
LP Enu Downregulated genes
LP Enu Upregulated genes
C
Biological processes for LG downregulated genes after Enu
Cholinergic synapse
Calcium signaling pathway
Long-term depression
p53 signaling pathway
ErbB signaling pathway
Biological processes for LG upregulated genes after Enu
Cholinergic synapse
GABAergic synapse
Glutamatergic synapse
Axon guidance
Long-term potentiation
Count
-log10 (p)
D
GO for LG Enu upregulated molecular pathways
Axon guidance
Glutamatergic synapse
AMPK signaling
Renin secretion
Long−term potentiation
Mucin type O−glycan biosynthesis
Phospholipase D signaling
cAMP signaling pathway
Relaxin signaling pathway
GnRH signaling
Retrograde endocannabinoid signaling
Aldosterone synthesis and secretion
Rap1 signaling pathway
Chemokine signaling
GO for LG Enu downregulated molecular pathways
Long−term depression
Gap junction
p53 signaling pathway
Purine metabolism
GO for LG Ctl and Enu common molecular pathways
Calcium signaling pathway
Oxytocin signaling pathway
Cholinergic synapse
Circadian entrainment
GABAergic synapse
Insulin secretion
ErbB signaling pathway
Molecular pathways
Enu downregulated gene
Enu upregulated gene
E
GO for Enu IN upregulated molecular pathways
Long−term depression
Gria1
Long−term potentiation
Ephb1
Axon guidance
Unc5c
Robo2
GO for Enu IN downregulated molecular pathways
Shc3
ErbB signaling pathway

Fig. S13. Molecular signature and gene ontology of modified by input deprivation. (A) Transcriptomic Euclidian distances between P3/P7, LG/LP and Ctl/Enu identities, based on the PCA space. (B) MA plot showing log fold-change (y-axis) compared with mean expression value (x-axis) (same filtering criteria as Fig. S9), negative fold change representing up-regulated genes in LG or LP Enu (light blue and light red respectively) and positive fold change representing down-regulated genes in LG or LP Enu (dark blue and dark red respectively). (C) Example of biological processes of gene ontologies associated with LG downregulated genes after enucleation (left) and LG upregulated genes after enucleation (right). (D) String plots representing gene ontology networks for LG Enu downregulated molecular pathways (dark blue), LG Enu upregulated molecular pathways (light blue) and LG Ctl and Enu common molecular pathways (purple). (E) String plots showing gene ontology networks for IN downregulated molecular pathways (dark red), IN Enu upregulated molecular pathways (light red). Ctl, control; Enu, Enucleation; IN, interneuron; LG, dorsolateral geniculate nucleus; LP, pulvinar/latero-posterior nucleus.

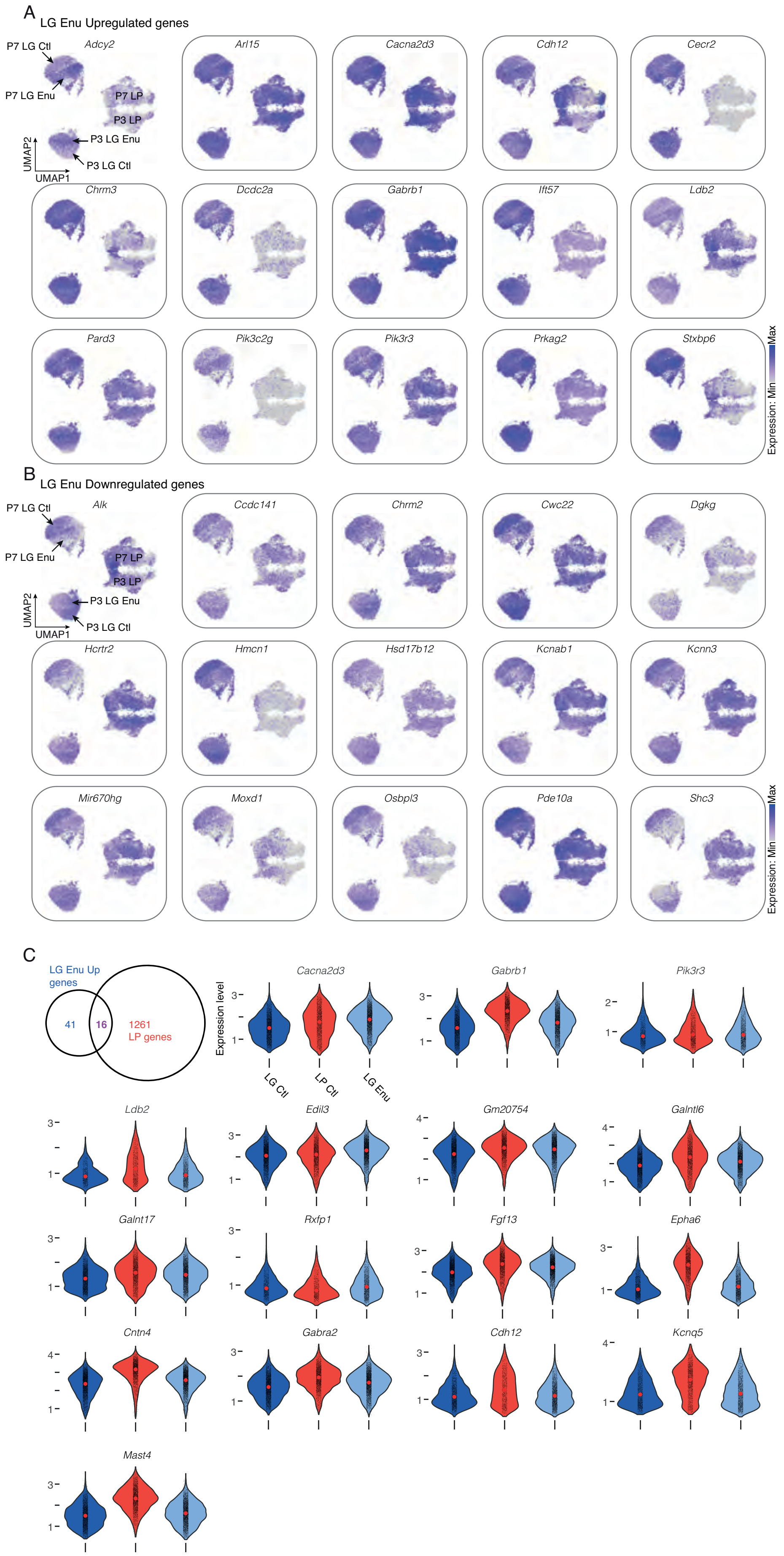

Development • Supplementary information

Fig. S14. Expression of select genes modified by input deprivation. (A) Example feature plot of select genes upregulated in LG after enucleation. (B) Example feature plot of select genes downregulated in LG after enucleation. (C) Venn diagram of overlapping genes among P7 LG Enu upregulated genes (n = 57) and P7 LP Ctl markers (n = 1277; Log Fold-change > 0.25). The expression of the 16 overlapping genes is represented using violin plot across the different groups, LG Ctl, LP Ctl and LG Enu (genes are highlighted in Table S16). Ctl, control; Enu, Enucleation; LG, dorsolateral geniculate nucleus.

Development • Supplementary information

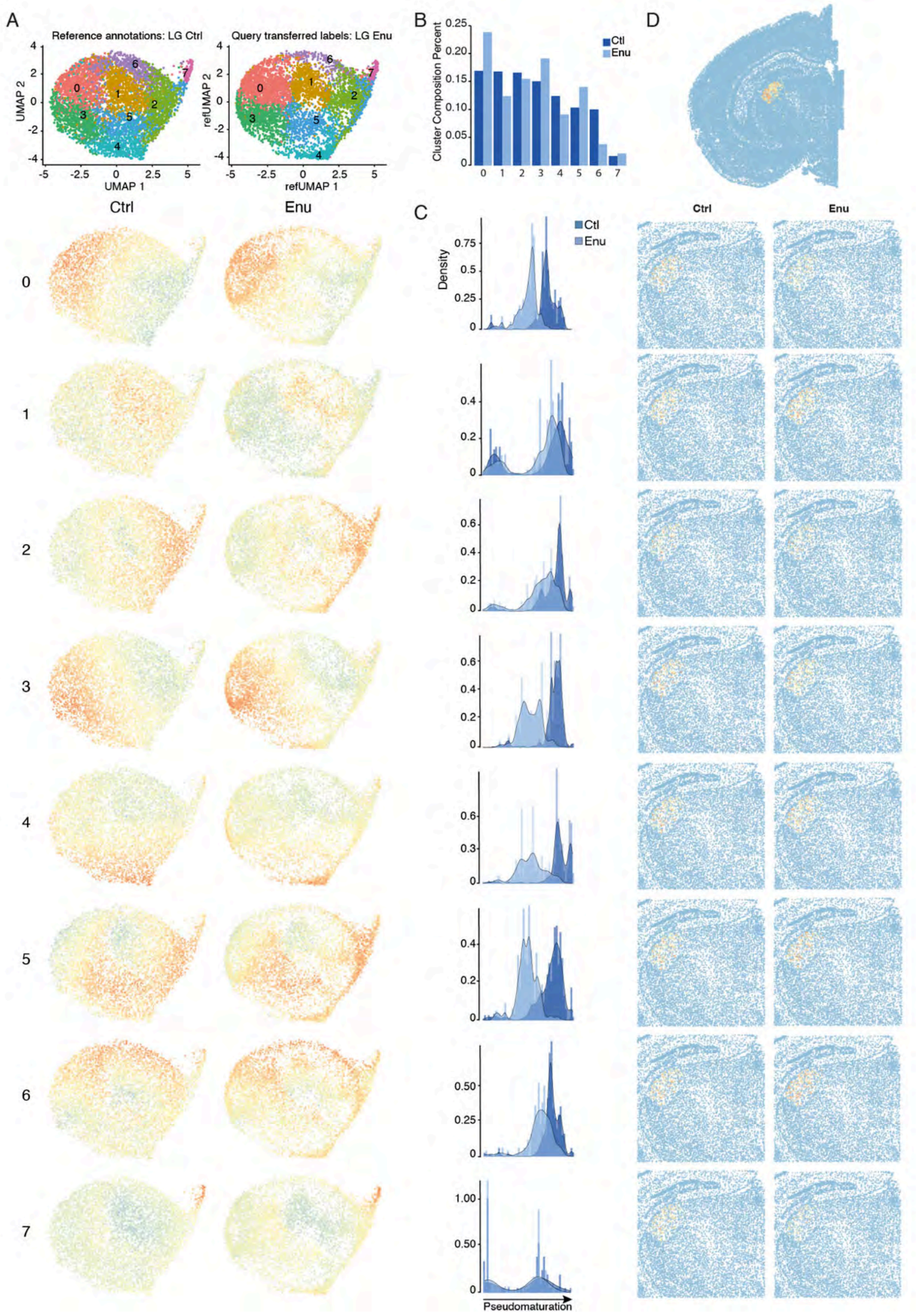

Fig. S15. Cell-type-specific transcriptional responses to input deprivation within LG. (A) UMAP representation of the integration of P7 LG Enu on the P7 LG Ctl embedding with unbiased clustering (see Methods). (B) Representation of cluster composition in Ctl and Enu condition displayed in a histogram. (C) Density of LG Ctl or LG Enu gene expression for each identified cluster along the pseudotime axis. (D) Spatial transcriptomic of differentially expressed genes of LG subclusters (spatial transcriptomic dataset from Zhang et al., 2023). Ctl, control; Enu, Enucleation; LG, dorsolateral geniculate nucleus.

Development • Supplementary information

Table S1. Apical progenitors, basal progenitors, early neurons and late neurons differentially expressed genes list (related to Fig. 1E).

Available for download at
https://journals.biologists.com/dev/article-lookup/doi/10.1242/dev.202764#supplementary-data

Table S2. Apical and basal cell cycle progenitors differentially expressed genes list (related to Fig. S5).

Available for download at
https://journals.biologists.com/dev/article-lookup/doi/10.1242/dev.202764#supplementary-data

Table S3. Apical progenitors, basal progenitors, early neurons and late neurons gene ontology analysis (related to Fig. 1F).

Available for download at
https://journals.biologists.com/dev/article-lookup/doi/10.1242/dev.202764#supplementary-data

Table S4. Gene expression across transcriptional waves along pseudotime (related to Fig. 2B).

Available for download at
https://journals.biologists.com/dev/article-lookup/doi/10.1242/dev.202764#supplementary-data

Table S5. Gene ontologies associated with transcriptional waves (related to Fig. 2C).

Available for download at
https://journals.biologists.com/dev/article-lookup/doi/10.1242/dev.202764#supplementary-data

Table S6. Comparison of developmental thalamic and cortical waves (related to Fig. 2B,C).

Available for download at
https://journals.biologists.com/dev/article-lookup/doi/10.1242/dev.202764#supplementary-data

Table S7. List of mature FO and HO genes used to define new differentially expressed genes (related to Fig. 3B).

Available for download at
https://journals.biologists.com/dev/article-lookup/doi/10.1242/dev.202764#supplementary-data

Table S8. FO and HO new differentially expressed genes and shared genes list (related to Fig. 3B).

Available for download at
https://journals.biologists.com/dev/article-lookup/doi/10.1242/dev.202764#supplementary-data

Table S9. FO and HO waves gene list (related to Fig. 3D).

Available for download at
https://journals.biologists.com/dev/article-lookup/doi/10.1242/dev.202764#supplementary-data

Table S10. List of FO and HO genes from Govek et al., 2022 (related to Fig. S8C).

Available for download at
https://journals.biologists.com/dev/article-lookup/doi/10.1242/dev.202764#supplementary-data

Table S11. Biological processes associated with FO and HO transcriptional maps clusters (related to Fig. S9).

Available for download at
https://journals.biologists.com/dev/article-lookup/doi/10.1242/dev.202764#supplementary-data

Table S12. List of mature VB, Po, LG and LP genes used to define new differentially expressed genes (related to Fig. 4A and B).

Available for download at
https://journals.biologists.com/dev/article-lookup/doi/10.1242/dev.202764#supplementary-data

Table S13. VB, Po, LG and LP new differentially expressed genes and shared genes list (related to Fig. 4A, B, G, H and I).

Available for download at
https://journals.biologists.com/dev/article-lookup/doi/10.1242/dev.202764#supplementary-data

Development • Supplementary information

Table S14. VB, Po, LG and LP waves gene list.

Available for download at
https://journals.biologists.com/dev/article-lookup/doi/10.1242/dev.202764#supplementary-data

Table S15. Biological processes associated with VB, Po, LG and LP transcriptional maps clusters (related to Fig. S11).

Available for download at
https://journals.biologists.com/dev/article-lookup/doi/10.1242/dev.202764#supplementary-data

Table S16. LG and LP Ctl vs. Enu differentially expressed genes list (related to Fig. 5B, S13 and S14).

Available for download at
https://journals.biologists.com/dev/article-lookup/doi/10.1242/dev.202764#supplementary-data

Table S17. Biological processes associated with LG Ctl, LG Enu, LP Ctl and LP Enu transcriptional maps clusters (related to Fig. S13).

Available for download at
https://journals.biologists.com/dev/article-lookup/doi/10.1242/dev.202764#supplementary-data

Table S18. P7 LG control subclusters marker genes and associated biological processes (related to Fig. S15).

Available for download at
https://journals.biologists.com/dev/article-lookup/doi/10.1242/dev.202764#supplementary-data

Table S19. P7 LG subclusters Ctl. Vs. Enu. differentially expressed genes list and associated biological processes (related to Fig. S15).

Available for download at
https://journals.biologists.com/dev/article-lookup/doi/10.1242/dev.202764#supplementary-data

Development • Supplementary information
